# Long-term neurological outcome after COVID-19 using all SARS-CoV-2 test results and hospitalisations in Denmark with 22-month follow-up

**DOI:** 10.1038/s41467-023-39973-6

**Published:** 2023-07-15

**Authors:** Clara S. Grønkjær, Rune H. B. Christensen, Daniel Kondziella, Michael E. Benros

**Affiliations:** 1 Biological and Precision Psychiatry, Copenhagen Research Center for Mental Health, Mental Health Centre Copenhagen, Copenhagen University Hospital, Copenhagen, Denmark; 2 Departments of Neurology, Rigshospitalet, Copenhagen University Hospital, Copenhagen, Denmark; 3 Department of Clinical Medicine, Faculty of Health and Medical Sciences, University of Copenhagen, Copenhagen, Denmark; 4 Department of Immunology and Microbiology, Faculty of Health and Medical Sciences, University of Copenhagen, Copenhagen, Denmark

**Keywords:** Neurological disorders, Viral infection

## Abstract

Hospitalisation with COVID-19 is associated with an increased risk of neurological sequelae; however, representative nationwide studies comparing to other infections with similar severity and also including milder SARS-CoV-2 infections have been lacking. Using the nationwide Danish registers including all SARS-CoV-2 PCR test results and hospitalisations between March 1, 2020, and December 31, 2021, we estimate the risk of any first neurological disorder diagnosed in inpatient, outpatient, or emergency room settings. We show that positive tests increase the rate of neurological disorders by a hazard ratio of 1.96 (95% confidence interval: 1.88–2.05) compared to individuals not tested and by a hazard ratio of 1.11 (95% confidence interval: 1.07-1.16) compared to individuals with negative tests only. However, there is no evidence that the risk of neurological disorders is higher for individuals who test positive compared to non-COVID-19 infections treated with anti-infective medication. The risk of neurological disorders is increased after COVID-19-hospitalisation compared to no COVID-19 hospital admission; however, these risks are comparable to hospitalisation with other respiratory infections (*P* value 0.328). In conclusion, COVID-19 is associated with an increased risk of neurological disorders, but no more than that observed after other infections of similar severity.

## Abstract (web summary)

Hospitalisation with COVID-19 is associated with an increased risk of neurological sequelae. Here in a nationwide Danish register study, the authors show that positive SARS-CoV-2 PCR tests and hospitalisations with COVID-19 are associated with increased risk of neurological sequelae, but with comparable risks as after other infections of similar severity.

## Introduction

The COVID-19 pandemic has exposed billions of people to an infection with SARS-CoV-2, with a yet undetermined risk of neurological sequelae. Due to the large number of infected individuals, the COVID-19 pandemic may have substantial consequences for brain health and pose public health challenges for neurorehabilitation. COVID-19 can lead to neurological symptoms, some of which are substantial and interfere with the ability to function in everyday life. These neurological sequelae may give rise to Long COVID, also termed *post-acute sequelae of SARS-CoV-2 infection* (PASC). However, three years into the pandemic the exact magnitude of the associations between COVID-19 and neurological disorders remains unknown.

Neurological manifestations in the acute phase of COVID-19 included headache, encephalopathy and stroke, which were present in more than 80% of hospitalised patients with COVID-19^1–4^, and the risk of neurological disorders remained increased after 3 months^5,6^. Compared with contemporary and historical control cohorts, patients with COVID-19 had an increased risk of cerebrovascular disorders, Alzheimer’s disease, peripheral nervous system disorders, myopathy, epilepsy, seizures, headaches, and migraines at 12-month follow-up^7,8^. The largest longitudinal COVID-19 study to date, based only on aggregated electronic health records from specific healthcare organisations, found that at 2-year follow-up, the risks of cognitive deficits, dementia, epilepsy, and seizures remained increased compared to other respiratory infections^9^. However, prior studies with long-term follow-up^10^ and adequate control groups were scarce and all were based on aggregate data from electronic health records without adjustment for important confounders of infectious and neurological diseases such as socio-economics, comorbidities for the past 40 years, and parental neurological disorders. Also, some studies compared pandemic data to pre-pandemic periods, which meant that the negative societal effects of lockdowns and changes in healthcare-seeking behaviour had not been accounted for^11^. Moreover, studies based on hospital records of exposure to SARS-CoV-2 only reflected the risk associated with severe COVID-19^12^. To our knowledge, no extensive nationwide register-based study that explores the neurological COVID-19 sequelae of an entire population has yet been conducted.

We conducted a nationwide register-based study using all SARS-CoV-2 PCR test results from the entire population of Denmark. We characterised the risk of neurological disorders through all new-onset neurological diagnoses made in inpatient and outpatient settings, including emergency room visits, over nearly two years after documented SARS-CoV-2 infection. We compared positive SARS-COV-2 tests with (1) individuals without tests, (2) individuals with only negative SARS-CoV-2 tests, and (3) individuals with other types of infections. We also explored whether the severity of COVID-19, as measured by admission to the hospital, including intensive care units (ICU), and the number of relapses, affected these risks over time. The risks were compared to the risks of neurological sequelae following other infections of similar severity and evaluated for different calendar periods and COVID-19 strains.

In this work, we show that a positive SARS-CoV-2 PCR test is associated with an increased risk of neurological disorders compared to both negative tests and no tests. Moreover, particularly hospitalisation with COVID-19 is associated with an increased risk of neurological sequelae. However, risks are comparable to the increased risks observed after other non-COVID infections of similar severity.

## Results

**Fig. 1:**
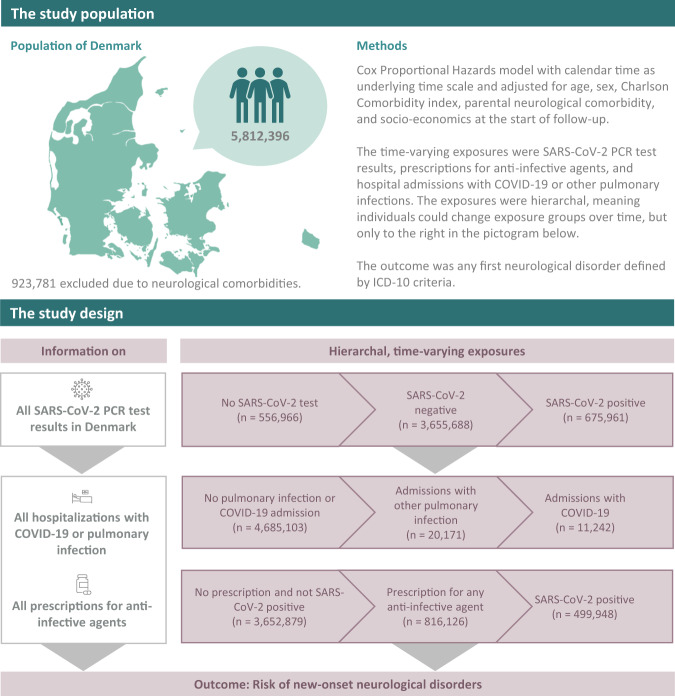
Schematic presentation of the study population, and study design. The study population (*n* = 4,888,615) is divided into three disjoint exposure groups in a hierarchical time-varying manner, where the same individual can contribute to observation time in all three exposure groups. Source data are provided as a Source Data file.

We identified 5,812,396 individuals living in Denmark from March 1, 2020, to December 31, 2021. We excluded 923,781 with a previous hospital contact for neurological disorders, therefore the study population consisted of 4,888,615 individuals (49.2% female, mean age at start (SD) 39.9 (23.3) years). During the study period, a total of 675,961 (13.8%) individuals tested positive for SARS-CoV-2, with 3,655,688 (74.8%) individuals only tested negative for SARS-CoV-2, and 556,966 (11.4%) individuals had no test for SARS-CoV-2 (Fig. 1 and Supplementary Table 1). A total of 12,545 (0.3%) were admitted to the hospital with COVID-19, of whom 1418 were admitted to an ICU during their hospital admission (Supplementary Table 2). A total of 89,013 (1.8%) were subsequently hospitalised for any neurological disorder, of which 2615 had previously tested positive for SARS-CoV-2.

### SARS-CoV-2 test results compared to the population not tested for SARS-CoV-2

Compared to the population not tested for SARS-CoV-2, individuals with a positive SARS-CoV-2 test had an elevated hazard ratio (HR) of 1.96 (95% confidence interval (CI) 1.88–2.05, *P* value < 0.001) for developing any first neurological disorder (Table 1). Individuals with a negative SARS-CoV-2 test had an increased HR of 1.76 (95% CI 1.73–1.79, *P* value < 0.001) compared to individuals with no test.

**Table 1:** Relative risks of any first neurological disorder for all individuals identified in Denmark, March 2020 to December 2021, by PCR test result, number of SARS-CoV-2 infections, time since SARS-CoV-2 infection, admission to hospital with COVID-19, and duration of COVID-19-admission

	Cases, No.	HR (95% CI)^a^	*P* value
SARS-CoV-2 PCR test			
No test	35,010	1.00 [reference]	–
Negative	51,388	1.76 (1.73–1.79)	<0.001
Positive	2615	1.96 (1.88–2.05)	<0.001
Positive vs. Negative	..	1.11 (1.07–1.16)	<0.001
Number of SARS-CoV-2 infections			
SARS-CoV-2 negative	51,388	1.00 [reference]	–
1	2596	1.11 (1.07–1.16)	<0.001
2+	19	1.83 (1.16–2.86)	0.009
Time since SARS-CoV-2 infection			
SARS-CoV-2 negative	51,388	1.00 [reference]	..
<1 month	367	1.04 (0.93–1.15)	0.502
1–2 months	497	1.05 (0.96–1.15)	0.268
3–5 months	669	1.18 (1.09–1.27)	<0.001
6–11 months	883	1.14 (1.07–1.22)	<0.001
12+ months	199	1.21 (1.05–1.39)	0.007
Hospital admission with COVID-19			
No COVID-19-admission^b^	88,577	1.00 [reference]	–
COVID-19-admission without ICU^c^	359	2.52 (2.27–2.79)	<0.001
COVID-19-admission with ICU^c^	77	5.64 (4.51–7.05)	<0.001
Readmissions with COVID-19			
No COVID-19-admission^b^	88,577	1.00 [reference]	–
1 admission	375	2.72 (2.46–3.02)	<0.001
2+ admissions	61	3.29 (2.56–4.23)	<0.001
Duration of COVID-19-admission			
No COVID-19-admission^b^	88,577	1.00 [reference]	..
1–2 bed days	93	2.56 (2.09–3.13)	<0.001
3–6 bed days	130	2.36 (1.98–2.80)	<0.001
7+ bed days	213	3.29 (2.88–3.77)	<0.001

The HRs with 95% CI and two-sided Wald *P* values unadjusted for multiple comparisons are from Cox Proportional Hazards models. Cases are the number of subjects with incident neurological disorders.

*HR* hazard ratio, *CI* confidence interval, *ICU* intensive care unit.

^a^Based on the Cox Proportional Hazards model stratified by age and adjusted for confounders (sex, parental neurology, Charlson Comorbidity Index, employment status, income, highest level of education).

^b^The reference group, no COVID-19-admission, consisted of individuals without admission to a hospital with SARS-CoV-2 infection, i.e., all individuals without PCR test results, with only negative test results, or a positive test result but no admission to hospital.

^c^The categorisation of COVID-19-admission and ICU is summarised in (Supplementary Table 20).

### SARS-CoV-2 positive test results and the risk of any first neurological disorder

**Fig. 2:**
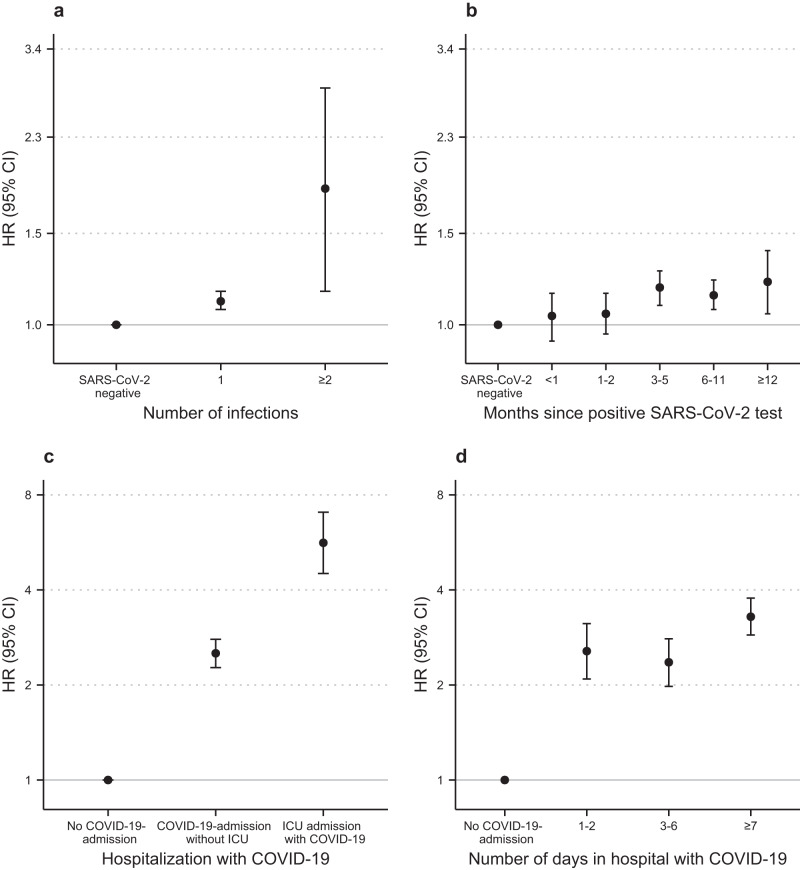
Risks of any first neurological disorder according to the severity of SARS-CoV-2 infection and at different time points since infection. Estimates are HRs with 95% CI from Cox Proportional Hazards models stratified by age and adjusted for confounders (sex, parental neurology, Charlson Comorbidity Index, employment status, income, and highest level of education). **a**, **b** Results are derived from a study population of *n* = 4,331,649 individuals with 54,003 cases of incident neurological disorders. **c**, **d** Results are derived from a study population of *n* = 4,888,615 individuals with 89,013 cases of incident neurological disorders. **a** Number of SARS-CoV-2 infections. **b** Time since infection. **c** Admission to hospital with COVID-19^ab^. **d** Duration of COVID-19-related admission^ab^. HR hazard ratio, CI confidence interval, ICU intensive care unit. Source data are provided as a Source Data file. ^a^The reference group, no COVID-19-admission, consisted of individuals without admission to a hospital with SARS-CoV-2 infection, i.e., all individuals without PCR test results, with only negative test results, or positive test results but no admission to hospital. ^b^The categorisation of admission to the hospital and ICU are summarised in Supplementary Table 20.

Individuals with a positive SARS-CoV-2 test had an increased HR of 1.11 (95% CI 1.07–1.16, *P* value < 0.001) for developing any first neurological disorder compared to individuals with a negative test (Table 1). The risk of neurological sequelae increased by the number of reinfections, where an isolated infection was associated with a slightly increased HR of 1.11 (95% CI 1.07–1.16, *P* value < 0.001), and two or more SARS-CoV-2 infections were associated with a higher HR of 1.83 (95% CI 1.16–2.86, *P* value 0.009) compared to individuals with only negative SARS-CoV-2 tests (Fig. 2). The risk remained increased even after one year.

**Fig. 3:**
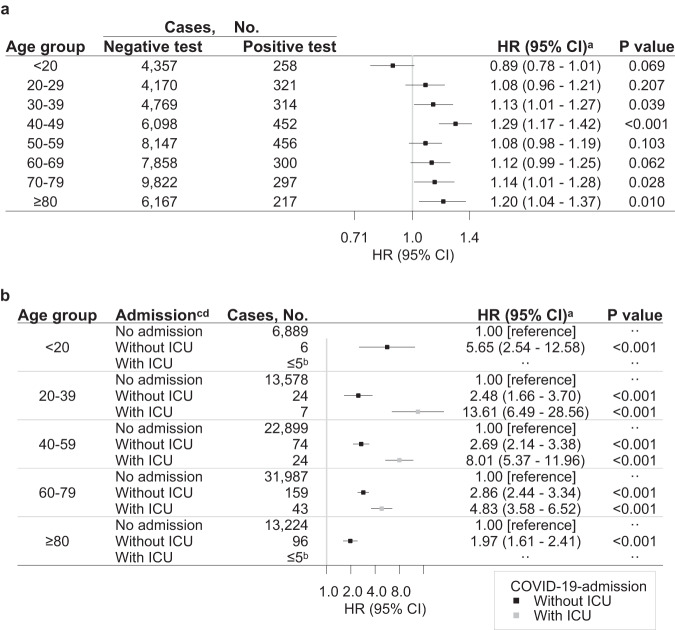
Risks of any first neurological disorder by age group and severity of SARS-CoV-2 infection. Results are derived from a study population of *n* = 4,888,615 individuals with 89,013 cases of incident neurological disorders. The HRs with 95% CI and two-sided Wald *P* values unadjusted for multiple comparisons are from Cox Proportional Hazards models. **a** Positive SARS-CoV-2 test compared to a negative SARS-CoV-2 test. **b** Admission to hospital with COVID-19 without ICU (black) and with ICU (grey) compared to no admission with COVID-19. HR hazard ratio, CI confidence interval. Source data are provided as a Source Data file. ^a^Based on a Cox Proportional Hazards model stratified by age and adjusted for confounders (sex, parental neurology, Charlson Comorbidity Index, employment status, income, and highest level of education). ^b^Results from ≤5 patients were omitted to ensure data privacy. ^c^The reference group, no COVID-19-admission, consisted of individuals without admission to a hospital with SARS-CoV-2 infection, i.e., all individuals without PCR test results, with only negative test results, or positive test results but no admission to hospital. ^d^The categorisation of admission to the hospital and ICU are summarised in Supplementary Table 20.

Additional analyses within different age groups showed that compared to SARS-CoV-2-negative individuals, a positive test was associated with a slightly decreased risk of neurological outcome for the youngest (<20 years), for whom the HR was 0.89 (95% CI 0.78–1.01, *P* value 0.069), whereas significantly increased risks were observed for the older age groups (30–49, and 70 to ≥80 years) (Fig. 3 and Supplementary Table 3–5).

**Fig. 4:**
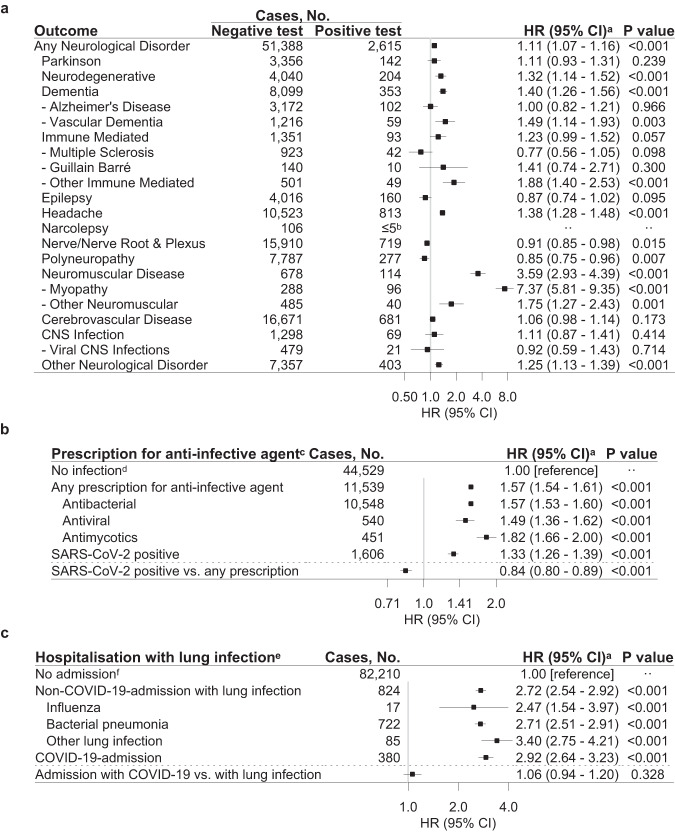
Specificity of risk of first neurological disorders after COVID-19 compared to other infections. Results are derived from a study population of *n* = 4,888,615 individuals with 89,013 cases of incident neurological disorders. The HRs with 95% CI and two-sided Wald *P* values unadjusted for multiple comparisons are from Cox Proportional Hazards models. **a** Positive SARS-CoV-2 test compared to a negative SARS-CoV-2 test. **b** Positive SARS-CoV-2 test and prescription for anti-infective agent compared to no infection. **c** Admission to hospital with COVID-19 or other pulmonary infections compared to no admission with COVID-19. HR hazard ratio, CI confidence interval. Source data are provided as a Source Data file. ^a^Based on a Cox Proportional Hazards model stratified by age and adjusted for sex, parental neurology, Charlson Comorbidity Index, employment status, income, and the highest level of education. ^b^Results from ≤5 patients were omitted to ensure data privacy. ^c^Excluding individuals with a redeemed prescription for any anti-infective agent between January 2019 and February 2020 to rule out recurring infections. The ATC codes for anti-infective agents are summarised in Supplementary Table 21. ^d^The reference group, no infection, consisted of all individuals without a prescription for an anti-infective agent and negative or no test result. ^e^Excluding individuals with any lung infection in a hospital between January 2010 and February 2020 to rule out recurring pulmonary infections. ICD-10 codes of pulmonary infection codes are summarised in Supplementary Table 22. ^f^The reference group, no admission, consisted of all individuals without admission to a hospital with SARS-CoV-2 infection or any pulmonary infection, i.e., all individuals not admitted to a hospital with pulmonary infection with either no test results or only negative test results, and individuals with a positive test result but no admission to hospital.

When comparing a positive COVID-19 test to a negative test, the neurological disorders with the highest HR were neuromuscular diseases (HR 3.59, 95% CI 2.93–4.39, *P* value < 0.001), in particular myopathy (HR 7.37, 95% CI 5.81–9.35, *P* value < 0.001; Fig. 4 and Supplementary Tables 6–8). Most neuromuscular cases are diagnosed with the ICD-10 code G72.9 (Myopathy, unspecified) according to the results with a higher granularity of diagnosis codes (Supplementary Table 9). Also, neurodegenerative diseases, dementia, vascular dementia, other immune-mediated disorders, headache, and other neurological disorders had increased HRs, whereas HRs were decreased for nerve/nerve root and plexus disorders, and polyneuropathy. The temporality of the risks depended on the nature of the neurological disorder. Within the first month after SARS-CoV-2 infection, the relative risk was greatest for Guillain–Barré syndrome (Supplementary Table 10 and Supplementary Fig. 1).

For positive SARS-CoV-2 test versus negative test, the risk of neurodegenerative diseases and dementia were increased in the elderly (60 to ≥80 years), including vascular dementia (≥80 years), immune-mediated disorders were at increased risk for the 40–79-year-olds, and headache for the 20–79 year-olds, whereas risks of neuromuscular disease and myopathy were increased in all age groups with a sufficient amount of cases (20–79 years; Supplementary Table 11 and Supplementary Fig. 2). The youngest age group (<20 years) did not have any specific disorder that was at decreased or increased risk.

### Hospitalisation with COVID-19

Hospitalisation with COVID-19 was associated with an increased HR of 2.52 (95% CI 2.27–2.79, *P* value < 0.001) for developing neurological disorders, compared to individuals without COVID-19-related hospital admission during the study period (Table 1 and Fig. 2). The risk of neurological sequelae increased in a dose-response relationship when measured by the number of admissions to the hospital and the duration of admission. The highest risks were for patients re-admitted to the hospital with COVID-19, where HR was 3.29 (95% CI 2.56–4.23, *P* value < 0.001), and patients in hospital at least 7 days, where HR was 3.29 (95% CI 2.88–3.77, *P* value < 0.001). ICU admission was associated with an even higher HR of 5.64 (95% CI 4.51–7.05, *P* value < 0.001) for developing neurological disorders, compared to individuals without COVID-19-related hospital admission during the study period.

The relative risk was elevated for all age groups, with the highest relative risk observed for the youngest age group (<20 years), where hospitalisation without ICU admission had an HR of 5.65 (95% CI 2.54-12.58, *P* value < 0.001) compared to no hospitalisation (Fig. 3 and Supplementary Table 12). The elevated relative risk became less pronounced with increasing age, and the smallest risk increase was observed in the oldest age group (≥80 years) having an HR of 1.97 (95% CI 1.61–2.41, *P* value < 0.001) for developing neurological disorders.

**Fig. 5:**
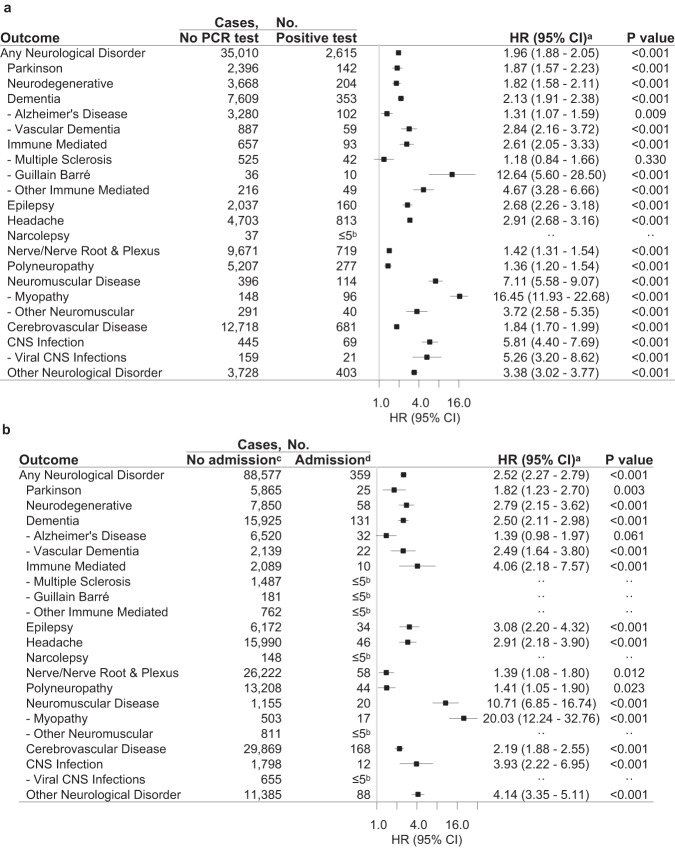
Risks of specific first neurological disorders after COVID-19 without and with admission to a hospital. Results are derived from a study population of *n* = 4,888,615 individuals with 89,013 cases of incident neurological disorders. The HRs with 95% CI and two-sided Wald *P* values unadjusted for multiple comparisons are from Cox Proportional Hazards models. **a** Positive SARS-CoV-2 test compared to no SARS-CoV-2 test. **b** Admission to a hospital with COVID-19 (without ICU admission) compared to no admission with COVID-19. HR hazard ratio, CI confidence interval. Source data are provided as a Source Data file. ^a^Based on a Cox Proportional Hazards model stratified by age and adjusted for confounders (sex, parental neurology, Charlson Comorbidity Index, employment status, income, highest level of education). ^b^Results from ≤5 patients were omitted to ensure data privacy. ^c^ The reference group, no COVID-19-admission, consisted of individuals without admission to a hospital with SARS-CoV-2 infection, i.e., all individuals without PCR test results, with only negative test results, or a positive test result but no admission to hospital. ^d^COVID-19-related admission without ICU admission (for exposure definitions, see Supplementary Table 20).

Hospitalised COVID-19 patients had a significantly increased risk of all neurological outcomes compared to non-hospitalised COVID-19 patients, except for Alzheimer’s disease (Fig. 5 and Supplementary Table 13). The outcome with the highest relative risk for hospitalised patients with COVID-19 was neuromuscular disease with an HR of 10.71 (95% CI 6.85–16.74, *P* value < 0.001) compared to individuals without COVID-19-related hospitalisation, and especially for myopathy, where HR was 20.03 (95% CI 12.24–32.76, *P* value < 0.001). After hospitalisation, the same pattern was observed for each age group as after a positive test, except for Dementia (≥80 years). Some age groups had increased risk of additional diseases; namely, vascular dementia and epilepsy (60–79 years), nerve/nerve root and plexus disorders and polyneuropathy (40–59 years), and cerebrovascular disease (40 to ≥80 years; Supplementary Table 14 and Supplementary Fig. 3).

COVID-19 patients admitted to the ICU also had increased relative risks for all ages and disorders where enough data were available (Supplementary Tables 13 and 15). The HR for patients admitted to the ICU compared to individuals without admission with COVID-19 was 61.34 (95% CI 35.35–106.42, *P* value < 0.001) for neuromuscular diseases, and 125.39 (95% CI 70.11–224.24, *P* value < 0.001) for myopathy.

### COVID-19 compared to other infections and the risk of neurological disorders

The risk of any neurological disorder associated with redeeming a prescription for any anti-infective agent was 1.57 (95% CI 1.54–1.61, *P* value < 0.001) compared to individuals without a prescription for anti-infective agents and negative or no test results (Fig. 4 and Supplementary Tables 16 and 17). However, the risk associated with COVID-19 was lower compared to individuals redeeming a prescription for any anti-infective agent, and HR was 0.84 (95% CI 0.80-0.89, *P* value < 0.001).

Any non-COVID-19 pulmonary infection treated in a hospital had increased HRs compared to the general population (Fig. 4 and Supplementary Table 17). However, the risk of COVID-19-related hospitalisations was similar to the risk increase observed after any hospital-treated non-COVID-19 pulmonary infection (1.06, 95% CI 0.94–1.20, *P* value 0.328). Analyses on subtypes of anti-infectives and pulmonary infections yielded similar results.

### Sensitivity Analysis

Several sensitivity analyses assessing the dependency on e.g., calendar periods did not change the main results and did not convey evidence of severe effect modification (see Supplementary material section on Sensitivity Analysis).

## Discussion

In this study of all SARS-CoV-2 PCR tests performed in Denmark between March 1, 2020, and December 31, 2021, and the subsequent risk of new-onset neurological disorders diagnosed in a hospital-setting, we found that a positive SARS-CoV-2 test was associated with a 96% increased rate of any neurological disorder when compared to individuals with no PCR test. However, when compared to individuals who tested negative for SARS-CoV-2, the rate of neurological disorders after a positive SARS-CoV-2 test was only elevated by 11%. Hospitalisation with COVID-19 was associated with a 2.5 times increased rate of neurological sequelae and 5.6 times higher with ICU admission. Importantly, risks increased in a dose-response relationship with reinfections and the number of admissions. Mild infections were not associated with increased risks for children and adolescents, but hospitalisation was. The risk differed for specific neurological disorders, being highest for neuromuscular diseases, dementia, headache, and neurodegenerative disorders. However, the risk of new-onset neurological disorders was decreased for individuals with COVID-19 compared to individuals redeeming a prescription for anti-infective agents. Moreover, the risk was similarly increased after hospitalisations for COVID-19 and non-COVID-19 hospital-treated pulmonary infections.

This study was the first to explore the dependency of reference groups by comparing positive tests to both negative and no tests. A previous study that compared positive to not positive individuals, i.e., individuals tested negative or not tested, found that the HR of neurological sequelae was 1.42 (95% CI 1.38–1.47) at 12-month follow-up^8^, which is in the range of our estimates, even though the previous study used US data where detection rates were low at the beginning of the pandemic^13^. Two previous studies also found that myopathy was the neurological disorder with the highest risk with an HR of 2.76 (95% CI 2.30–3.32)^8^ for positive compared with not positive, and 6.02 (95% CI 3.77–9.62, *P* value < 0.0001)^10^ for COVID-19 compared with influenza.

Our study supported findings on an increased risk of neurological disorders after COVID-19-related hospitalisation^5^. One study estimated the relative risk of neurological sequelae (including mental disorders, prescription records, and laboratory tests) to 2.87 (95% CI 2.57-3.22) for hospitalised patients with COVID-19 compared to not positive patients without hospitalisation at 12-month follow-up, and 4.00 (95% CI 3.02-5.31) for patients in ICU^8^. Another study estimated the risk of COVID-19-related hospitalisation to 1.70 (95% CI 1.56-1.86, *P* value < 0.0001) compared to individuals with COVID-19 not admitted to the hospital, and admission to the ICU was associated with an increased risk of 2.87 (95% CI 2.45-3.35, *P* value < 0.0001)^10^. Although previous studies found that COVID-19-related hospitalisation had a higher risk of neurological disorders that subsided after two years compared with influenza and other respiratory infections treated in hospitals^9,10^, we found no difference. However, these prior studies used aggregate data with unknown record completeness, without prior diagnosis validation having been done, little socioeconomic information, and no information before the first hospital contact^9,10^. Moreover, clinical studies have indicated that there is a comparable burden of neuropsychiatric diagnoses after hospitalisation for COVID-19 compared to matched controls with hospitalisations due to non-COVID-19 causes^14^.

The contagiousness and severity of SARS-CoV-2, sequelae of COVID-19, and people’s testing behaviour are likely affected by the evolving pandemic with new SARS-CoV-2 variants, lock-down measures, testing strategies, vaccinations^15^, and therapeutics for severe acute COVID-19. Many elderlies were never tested, whereas younger people got tested more often out of necessity to participate in social life and were tested without symptoms, hence a positive test in the younger age group may indicate very mild infections (and sometimes even false positives). However, the risk estimates did not significantly change over time. Nonetheless, individuals with no testing for SARS-CoV-2 might not seek medical attention at the same rate as tested individuals, which could have impacted the results. Thus, the risk estimates compared to the no-test reference group might be falsely high and should be interpreted with caution. We consider the comparison between positive and negative tests to be most meaningful for interpreting and communicating the impact of SARS-CoV-2 on neurological sequelae.

The major strengths of this study are as follows: First, we utilised the well-validated, nationwide Danish registers that allowed for analyses of the entire population, including people of all ages with full information on exposure (PCR test, hospitalisation), outcomes (neurological disorders diagnosed in an inpatient or outpatient setting, including emergency room visits), and important individual covariates such as age, sex, comorbidity, socioeconomics, and similar data for individuals’ parents). Second, the Danish registers are well-known for their high validity regarding the main diagnostic disease categories^16–24^, thus the results from this nationwide study are representative of the population of Denmark. Third, the models were adjusted for important confounders and the results were robust also in the sensitivity analyses. Fourth, we investigated an exhaustive list of prespecified neurological outcomes in the acute phase of COVID-19 to 22-month follow-up. Last, extensive testing strategies set up by the Danish government meant that more than 80% of the population was tested for SARS-CoV-2 infection during the pandemic, facilitating suitable and contemporary control groups and reducing misclassification bias. Although PCR tests have high false negative rates and are biased towards specific groups in the population, they are still the best detector for SARS-CoV-2^25,26^. Moreover, the numbers of COVID-19 tests, test results, and admissions were consistent with the official Danish numbers^27^.

The limitations of this study firstly included a potential bias due to the considerable amount of attention to COVID-19, which could result in healthcare workers being more attentive to possible sequelae among COVID-19 survivors. As another limitation, we only included neurological disorders diagnosed in an inpatient or outpatient setting, including emergency room visits, thereby potentially missing less severe cases of neurological sequelae such as headache and migraine, which are typically treated in primary care facilities. Lastly, individuals were followed up to 22 months, which adequately estimated medium-term risks, but longer follow-up times are desired. However, expanding the time frame comes at the cost of introducing surveillance bias since the number of conducted tests dropped drastically during 2022 with the use of at-home rapid tests.

In conclusion, COVID-19 is associated with an increased risk of developing neurological disorders, particularly neuromuscular diseases, and increased risks are also observed in people with COVID-19 who avoid hospitalisation. Although risks differ between age groups and specific disorders, these risks increase with the severity of COVID-19 and the number of infections in a dose-dependent manner. Importantly, however, risks are comparable to the increased risks observed in individuals hospitalised for non-COVID-19 pulmonary infections or with non-COVID-19 infections treated with prescription medication for anti-infective agents. This suggests that COVID-19 neurological sequelae are not intrinsically different from the neurological sequelae seen after non-COVID-19 infections of similar severity.

## Methods

### Study population

We conducted a nationwide population-based, register-linked, observational study by identifying all individuals alive on March 1, 2020, within the entire population of Denmark (5.8 million inhabitants). In the Danish Civil Registration System^28^ information on age, sex, hospital contacts, prescription data, linkage to parents, and socioeconomics was provided for every resident, and a unique personal identification number enabled complete linkage among the Danish registers. We had information available from the registers up to December 31, 2021. Individuals were followed from March 1, 2020, until the onset of the disorders of interest, and censored in case of emigration, death, or end of follow-up on December 31, 2021, whichever came first. This study was approved by the Danish Data Protection Agency and the Danish Health and Medicine Authority. According to Danish legislation, no further ethical approval or informed consent is required for register-based studies.

### Exposure to SARS-CoV-2

By taking advantage of the extensive testing facilities established by the Danish government, we identified all individuals who tested negative and positive for SARS-CoV-2 since the start of the pandemic and over the course of nearly two years in Denmark. We defined confirmed COVID-19 as having a positive SARS-CoV-2 polymerase chain reaction (PCR) by nasopharyngeal/tracheal test result. Test results were extracted from The Microbiology Database (MiBa)^29^, which contained all COVID-19 test results in Denmark between February 2, 2020, and March 10, 2022. There is free access to health care in Denmark, and COVID-19 tests were free and easily available, with equal access to testing facilities^30^.

### Assessment of neurological outcomes

Information on neurological disorders was included from the Danish National Patient Register coded according to the *International Classification of Diseases, Eighth Revision (ICD-8)* from 1977 and *Tenth Revision (ICD-10)* from 1994 onwards. For dementia specifically, the relevant codes from the Danish Psychiatric Central Research Register^31,32^ were also used (Supplementary Table 18). The codes were assigned by the treating clinician who discharged the patient, and afterwards, the data were automatically checked and returned to the source hospital in case of errors^16^.

#### Primary outcome

We identified neurological sequelae of COVID-19 in terms of any first-time diagnoses of a neurological disorder (ICD-10: A066, A17, A321, A390, A521-A523, A80-A89, B003-B004, B010-B011, B020-B021, B050-B051, B060, B261-B262, B375, B451, B582, E236A, F00-F03, G00-G99, I60-69, M35.0, M32, M05-M06, M08.0) from inpatient, outpatient, or emergency room contacts. The date of illness onset was defined as the first day of the first hospital contact. We omitted all ICD-10 diagnoses with the codes suspected or not found.

#### Secondary outcomes

A first diagnosis within the following specific categories: Parkinson’s disease and parkinsonism, neurodegenerative diseases, dementia (of any kind), Alzheimer’s disease, vascular dementia, immune-mediated, Multiple Sclerosis, Guillain–Barré syndrome, other immune-mediated, epilepsy, headache, narcolepsy, nerve/nerve root and plexus disorders, polyneuropathy, neuromuscular disease, myopathy, other neuromuscular diseases, cerebrovascular disease, central nervous system (CNS) infections, viral CNS infections, and other neurological disorders (Supplementary Table 18)^33,34^.

### Statistical analysis

We performed Cox Proportional Hazards regression with calendar time as the underlying time scale and stratified by age at the start of the follow-up, which was March 1, 2020. We reported hazard ratios (HRs) including 95% Confidence Intervals (CIs). Statistical analyses were done in R, version 4.1.3 with the survival package version 3.2–13, and the statistical significance was set to a two-sided *P* value < 0.05 (see Supplementary Methods Section for more details).

All analyses were adjusted for the following established and suspected risk factors for COVID-19 and neurological diagnoses: Age, sex, Charlson Comorbidity Index (CCI)^35^ (Supplementary Table 19), any parental neurological history (Supplementary Table 18), and socioeconomic factors (employment status, income quantile^36^, and educational attainment level) at start of follow-up.

For each outcome diagnosis, we excluded patients who already had the diagnosis in question from a hospital before the start of follow-up (e.g., when investigating epilepsy, we excluded patients diagnosed with epilepsy before the start of follow-up, but not patients with other neurological diagnoses such as migraine; see Supplementary Table 18).

#### The primary analyses

compared three groups: (1) individuals with a positive SARS-CoV-2 test, (2) individuals with only negative SARS-CoV-2 tests, and (3) individuals without any PCR test result. PCR tests were identified in a hierarchal time-varying manner, meaning everyone started in the no test group and moved to the negative or positive group depending on test results. In the positive group, subsequent negative tests were ignored.

#### In the secondary analyses

we investigated whether the neurological sequelae of COVID-19 were affected by the severity of the illness, as measured by (1) the number of COVID-19 relapses (if any), (2) hospital admission, (3) the number of days in the hospital, (4) the number of admissions, and (5) ICU admission (for exposure definitions see Supplementary Table 20)^37–39^. The trajectory of the illness was assessed by measuring outcomes for five subgroups: <1 month, 1–2 months, 3–5 months, 6–11 months, and more than 12 months after infection.

To provide benchmarks for the risk of neurological sequelae, people who had suffered from COVID-19 were compared to people with non-COVID-19 pulmonary infections, which was done separately for hospital and not hospital-treated infections. More specifically, for infections not treated in hospitals, we compared SARS-CoV-2 positive tests to (1) any prescription of anti-infective agents, and (2) subtypes of anti-infectives (antibacterial, antiviral, and antimycotic agents) (for ATC codes see Supplementary Table 21)^40^, identified using The Danish National Prescription Register, which contained information on all redeemed prescriptions since 1995 grouped by ATC codes^41^. For hospital-treated infections, we compared COVID-19-admission with (1) any pulmonary infection, and (2) subtypes of pulmonary infections (influenza, bacterial pneumonia, and other pulmonary infections) (for diagnosis codes see Supplementary Table 22), identified using the Danish National Patient Register.

### Sensitivity analysis

In sensitivity analyses, we assessed the robustness of results by repeating the analysis with various adjustments for confounders. Also, effect modification by immigration status was performed, since the Danish registers do not include information before immigration, i.e., pre-existing neurological conditions and past infections are unknown. Since some neurological disorders are diagnosed in non-hospital facilities, we also analysed effect modification according to whether individuals had redeemed a prescription for a pre-existing neurological disorder before the start of follow-up (Supplementary Table 18). To search for possible bias related to calendar time, we looked at the number of conducted tests at the individual level, periods with lock-down, and different SARS-CoV-2 variants.

### Reporting summary

Further information on research design is available in the Nature Portfolio Reporting Summary linked to this article.

## Supplementary information

Supplementary Information

Peer Review File

Reporting Summary

## Data Availability

The datasets analysed in the current study were the Microbiology Database, the Danish National Patient Register, the Danish Psychiatric Central Research Register, the Population Education Register, the Income Statistics Register, and the Danish National Prescription Register. The data used in the study are available from Statistics Denmark, https://www.dst.dk/en/TilSalg/Forskningsservice/Dataadgang. Applications to access health data in Denmark are submitted to the Danish Data Protection Agency, the Danish National Board of Health and Statistics Denmark. Information can be found at https://www.itgovernance.eu/da-dk/eu-gdpr-compliance-dk, https://sundhedsdatastyrelsen.dk/da/english, and https://dst.dk/en. Source data are provided with this paper.

## Source data

Source data

## Code availability

The data collection, analyses, and illustrations were made in R version 4.1.3. The Cox Proportional Hazards Analyses were conducted using the function *coxph* from the survival package version 3.2–13.
