# Supplementary material for: Long-term neurological outcome after COVID-19 using all SARS-CoV-2 test results and hospitalisations in Denmark with 22-month follow-up
Source: Nat Commun. 2023 Jul 15;14:4235. doi: 10.1038/s41467-023-39973-6 (PMC10349860; doi:10.1038/s41467-023-39973-6)
Supplement: Supplementary file 1 — Supplementary Information [file 41467_2023_39973_MOESM1_ESM.pdf]

Supplementary Material

Cohort Characteristics **3**

Supplementary Table 1: Characteristics of the Study Population for each of the following categories, no SARS-CoV-2 PCR test, negative SARS-CoV-2 PCR test, and positive SARS-CoV-2 PCR test. *3*

Supplementary Table 2: Characteristics of the Study Population divided into no hospitalisation and hospitalisation with and without ICU admission *5*

Results: Positive SARS-CoV-2 test **7**

Supplementary Table 3: Risks of any first neurological disorder following a COVID-19 negative test compared to no test across age groups *7*

Supplementary Table 4: Risks of any first neurological disorder following a positive SARS-CoV-2 test compared to no SARS-CoV-2 test across age groups *7*

Supplementary Table 5: Risks of any first neurological disorder following a positive SARS-CoV-2 test compared to a negative SARS-CoV-2 test across age groups *8*

Supplementary Table 6: Risk of specific first neurological outcomes among individuals with negative SARS-CoV-2 test compared to no SARS-CoV-2 test *9*

Supplementary Table 7: Risks of specific first neurological outcomes among individuals with a positive SARS-CoV-2 test compared to no SARS-CoV-2 test *10*

Supplementary Table 8: Risks of specific first neurological outcomes among individuals with a positive SARS-CoV-2 test compared to a negative SARS-CoV-2 test *11*

Supplementary Table 9: Risks of specific first polyneuropathic and neuromuscular outcomes among individuals with a positive SARS-CoV-2 test compared to a negative SARS-CoV-2 test *12*

Supplementary Table 10: Risks of specific first neurological outcomes at different time points since the first SARS-CoV-2 positive test compared with SARS-CoV-2 negative tests *13*

Supplementary Figure 1: Plot of the HR and 95% CI for the risks of specific first neurological outcomes at different time points since the first SARS-CoV-2 positive test compared to a negative test. *16*

Supplementary Table 11: Risks of specific first neurological outcomes among SARS-CoV-2 positive individuals compared with SARS-CoV-2 negative individuals across age groups *17*

Supplementary Figure 2: Forest plot of the association between SoV-2 positive test result and specific neurological outcomes across age groups compared to SARS-CoV-2 negative test result *20*

Results: Hospitalisation **21**

Supplementary Table 12: Risks of any first neurological disorder following COVID-19-related hospitalisation with and without ICU admission compared to no COVID-19-related hospitalisation across age groups *21*

Supplementary Table 13: Risks of specific neurological disorders for COVID-19-related hospitalisation with and without ICU admission compared to individuals without admission to a hospital with SARS-CoV-2 infection *22*

Supplementary Table 14: Specific neurological disorders for COVID-19-related hospitalisations without admission to ICU compared to individuals without admission to a hospital with COVID-19 across age groups *23*

Supplementary Figure 3: Forest plot of the risks associated with admissions to hospital with COVID-19 and specific neurological outcome across age groups compared to individuals without hospital admission with COVID-19 .... *26*

Supplementary Table 15: Specific neurological disorders for hospitalised individuals with COVID-19 with ICU admission compared to individuals without COVID-19-admission across age groups *27*

Supplementary Table 16: Number of individuals with other infections *28*

Supplementary Table 17: Specificity of risks associated with SARS-CoV-2 compared to any prescription for anti-infective agents, and of COVID-19 hospitalisation compared to non-COVID-19 lung infection treated in hospital *29*

Sensitivity Analysis **30**

Sensitivity analysis 1: Various levels of adjusting for confounders *30*

Sensitivity analysis 2: Immigration status *30*
Sensitivity analysis 3: Prescription for a pre-existing neurological disorder *31*
Sensitivity analysis 4: Number of SARS-CoV-2 PCR tests *31*
Sensitivity analysis 5: Effect of lockdown *32*
Sensitivity analysis 6: Effect of virus variants *32*

Overview of ICD-8, ICD-10, and ATC codes 33
Supplementary Table 18: Neurological disorders categorized by ICD-8, ICD-10, and ATC codes *33*
Supplementary Table 19: Charlson Comorbidity Index categorized by ICD-8 and ICD-10 codes *34*
Supplementary Table 20: Definition of Exposures *35*
Supplementary Table 21: Anti-infective agents categorized by ATC codes[10] *35*
Supplementary Table 22: Infection codes categorized by ICD-10 codes *35*
Supplementary Table 23: Comorbidities related to neurological disorders and COVID-19 categorized by ICD-8 and ICD-10 codes *36*

Supplementary Methods 37

STROBE Statement 38
STROBE Checklist: Items that should be included in reports of cohort studies *38*

References 40

Cohort Characteristics

Supplementary Table 1: Characteristics of the Study Population for each of the following categories, no SARS-CoV-2 PCR test, negative SARS-CoV-2 PCR test, and positive SARS-CoV-2 PCR test.

	Total	SARS-CoV-2 positive[a]	SARS-CoV-2 negative[b]	No PCR test[c]
**Total at end of follow-up (% by row)**	4,888,615 (100%)	675,961 (13.8%)	3,655,688 (74.8%)	556,966 (11.4%)
**Neurological disorder at end of follow up (% by column)**				
Yes	89,013 (1.8%)	2,615 (0.4%)	51,388 (1.4%)	35,010 (6.3%)
No	4,799,602 (98.2%)	673,346 (99.6%)	3,604,300 (98.6%)	521,956 (93.7%)
**Time of Inclusion in Exposure Group (% by column)**				
2020, March – 2020, June	1,137,281 (23.3%)	9,819 (1.5%)	570,496 (15.6%)	556,966 (100.0%)
2020, July – 2020, December	2,286,211 (46.8%)	131,221 (19.4%)	2,154,990 (58.9%)	..
2021, January – 2021, June	769,397 (15.7%)	105,265 (15.6%)	664,132 (18.2%)	..
2021, July – 2021, December	695,726 (14.2%)	429,656 (63.6%)	266,070 (7.3%)	..
	No. individuals (% by column) by the start of follow up			
**Mean Age (SD)**	39.2 (23.3)	31.5 (19.4)	38.8 (22.8)	51.1 (26.0)
**Age group**				
<20	1,239,431 (25.4%)	226,510 (33.5%)	929,799 (25.4%)	83,122 (14.9%)
20-29	709,280 (14.5%)	128,097 (19.0%)	524,332 (14.3%)	56,851 (10.2%)
30-39	600,156 (12.3%)	95,009 (14.1%)	460,080 (12.6%)	45,067 (8.1%)
40-49	629,068 (12.9%)	94,786 (14.0%)	485,324 (13.3%)	48,958 (8.8%)
50-59	637,896 (13.0%)	73,304 (10.8%)	496,234 (13.6%)	68,358 (12.3%)
60-69	500,323 (10.2%)	34,711 (5.1%)	379,312 (10.4%)	86,300 (15.5%)
70-79	401,019 (8.2%)	17,720 (2.6%)	278,269 (7.6%)	105,030 (18.9%)
≥80	171,442 (3.5%)	5,824 (0.9%)	102,338 (2.8%)	63,280 (11.4%)
**Sex**				
Female	2,404,186 (49.2%)	334,885 (49.5%)	1,821,281 (49.8%)	248,020 (44.5%)
Male	2,484,429 (50.8%)	341,076 (50.5%)	1,834,407 (50.2%)	308,946 (55.5%)
**Mean Duration in Years (SD)**	1.8 (0.2)	1.8 (0.1)	1.8 (0.2)	1.7 (0.5)
**Parental Neurological Disorder**				
Yes	1,577,633 (32.3%)	227,175 (33.6%)	1,219,846 (33.4%)	130,612 (23.5%)
No	3,310,982 (67.7%)	448,786 (66.4%)	2,435,842 (66.6%)	426,354 (76.5%)
**Charlson Comorbidity Index**				
0	4,022,678 (82.3%)	587,666 (86.9%)	3,007,923 (82.3%)	427,089 (76.7%)
1	409,989 (8.4%)	52,526 (7.8%)	310,537 (8.5%)	46,926 (8.4%)
2	277,523 (5.7%)	23,504 (3.5%)	206,222 (5.6%)	47,797 (8.6%)
3+	178,425 (3.6%)	12,265 (1.8%)	131,006 (3.6%)	35,154 (6.3%)
**Education**				
No education	891,117 (18.2%)	149,709 (22.1%)	649,334 (17.8%)	92,074 (16.5%)
Primary school	1,147,215 (23.5%)	153,208 (22.7%)	827,196 (22.6%)	166,811 (29.9%)
Vocational training or gymnasium	1,555,383 (31.8%)	197,895 (29.3%)	1,171,964 (32.1%)	185,524 (33.3%)
Higher education, short cycle	777,795 (15.9%)	100,906 (14.9%)	604,624 (16.5%)	72,265 (13.0%)
Higher education, long cycle	517,105 (10.6%)	74,243 (11.0%)	402,570 (11.0%)	40,292 (7.2%)
**Employment status**				
Kids and Education	1,468,348 (30.0%)	272,102 (40.3%)	1,103,933 (30.2%)	92,313 (16.6%)
Employed	2,355,653 (48.2%)	338,960 (50.1%)	1,820,003 (49.8%)	196,690 (35.3%)

Not in workforce	168,065 (3.4%)	19,091 (2.8%)	122,669 (3.4%)	26,305 (4.7%)
Retired	842,876 (17.2%)	38,869 (5.8%)	572,908 (15.7%)	231,099 (41.5%)
Unemployed or unknown	53,673 (1.1%)	6,939 (1.0%)	36,175 (1.0%)	10,559 (1.9%)
**Income Quantile**				
0-20%	1,065,476 (21.8%)	178,504 (26.4%)	778,048 (21.3%)	108,924 (19.6%)
20-40%	936,009 (19.1%)	141,890 (21.0%)	669,336 (18.3%)	124,783 (22.4%)
40-60%	873,090 (17.9%)	88,880 (13.1%)	621,076 (17.0%)	163,134 (29.3%)
60-80%	977,120 (20.0%)	121,431 (18.0%)	761,415 (20.8%)	94,274 (16.9%)
80-100%	1,036,920 (21.2%)	145,256 (21.5%)	825,813 (22.6%)	65,851 (11.8%)
**Autoimmune Disease**				
Yes	223,216 (4.6%)	24,896 (3.7%)	170,402 (4.7%)	27,918 (5.0%)
No	4,665,399 (95.4%)	651,065 (96.3%)	3,485,286 (95.3%)	529,048 (95.0%)
**Heart Disease**				
Yes	554,631 (11.3%)	43,181 (6.4%)	409,072 (11.2%)	102,378 (18.4%)
No	4,333,984 (88.7%)	632,780 (93.6%)	3,246,616 (88.8%)	454,588 (81.6%)
**Trauma within two years**				
Yes	182,574 (3.7%)	26,793 (4.0%)	138,217 (3.8%)	17,564 (3.2%)
No	4,706,041 (96.3%)	649,168 (96.0%)	3,517,471 (96.2%)	539,402 (96.8%)

Abbreviations: SD, Standard Deviation

Table description: The exposure groups, *SARS-CoV-2 positive PCR test*, *SARS-CoV-2 negative PCR test*, and *no SARS-CoV-2 PCR test*, were identified by the end of follow up

[a] The group *SARS-CoV-2 positive PCR test* consisted of everyone with at least one positive test by the end of follow up
[b] The group *SARS-CoV-2 negative PCR test* consisted of everyone with at least one negative and no positive test by the end of follow up
[c] The group *No SARS-CoV-2 PCR test* consisted of everyone without any PCR test result by the end of follow up

Supplementary Table 2: Characteristics of the Study Population divided into no hospitalisation and hospitalisation with and without ICU admission

	No COVID-19 hospitalisation[a]	COVID-19-hospitalisation without ICU[b]	COVID-19-hospitalisation with ICU[c]
**Total at end of follow-up (% by row)**	4,876,070 (99.74%)	11,127 (0.23%)	1,418 (0.03%)
**Neurological disorder at end of follow up (% by column)**			
Yes	88,577 (1.8%)	359 (3.2%)	77 (5.4%)
No	4,787,493 (98.2%)	10,768 (96.8%)	1,341 (94.6%)
**Time of Inclusion in Exposure Group**			
2020, March – 2020, June	4,876,070 (100.0%)	1,352 (12.2%)	234 (16.5%)
2020, July – 2020, December	..	2,946 (26.5%)	338 (23.8%)
2021, January – 2021, June	..	3,388 (30.4%)	448 (31.6%)
2021, July – 2021, December	..	3,441 (30.9%)	398 (28.1%)
	**No. individuals (% by column) by the start of follow up**		
**Mean Age (SD)**	39.2 (23.2)	56.0 (21.8)	61.2 (16.7)
**Age group**			
<20	1,238,911 (25.4%)	490 (4.4%)	30 (2.1%)
20-39	1,306,830 (26.8%)	2,470 (22.2%)	136 (9.6%)
40-59	1,263,515 (25.9%)	3,049 (27.4%)	400 (28.2%)
60-79	897,212 (18.4%)	3,389 (30.5%)	741 (52.3%)
≥80	169,602 (3.5%)	1,729 (15.5%)	111 (7.8%)
**Sex**			
Female	2,398,341 (49.2%)	5,394 (48.5%)	451 (31.8%)
Male	2,477,729 (50.8%)	5,733 (51.5%)	967 (68.2%)
**Mean Duration in Years (SD)**	1.8 (0.2)	1.7 (0.3)	1.5 (0.5)
**Parental Neurological Disorder**			
Yes	1,574,559 (32.3%)	2,765 (24.8%)	309 (21.8%)
No	3,301,511 (67.7%)	8,362 (75.2%)	1,109 (78.2%)
**Charlson Comorbidity Index**			
0	4,015,454 (82.4%)	6,510 (58.5%)	714 (50.4%)
1	408,316 (8.4%)	1,469 (13.2%)	204 (14.4%)
2	275,955 (5.7%)	1,358 (12.2%)	210 (14.8%)
3+	176,345 (3.6%)	1,790 (16.1%)	290 (20.5%)
**Education**			
No education	890,218 (18.3%)	791 (7.1%)	108 (7.6%)
Primary school	1,143,404 (23.4%)	3,378 (30.4%)	433 (30.5%)
Vocational training or gymnasium	1,550,741 (31.8%)	4,064 (36.5%)	578 (40.8%)
Higher education, short cycle	775,703 (15.9%)	1,888 (17.0%)	204 (14.4%)
Higher education, long cycle	516,004 (10.6%)	1,006 (9.0%)	95 (6.7%)
**Employment status**			
Kids and Education	1,467,488 (30.1%)	816 (7.3%)	44 (3.1%)
Employed	2,350,288 (48.2%)	4,824 (43.4%)	541 (38.2%)
Not in workforce	167,240 (3.4%)	734 (6.6%)	91 (6.4%)
Retired	837,564 (17.2%)	4,586 (41.2%)	726 (51.2%)
Unemployed or unknown	53,490 (1.1%)	167 (1.5%)	16 (1.1%)
**Income Quantile**			

0-20%	1,064,953 (21.8%)	479 (4.3%)	44 (3.1%)
20-40%	933,327 (19.1%)	2,351 (21.1%)	331 (23.3%)
40-60%	868,894 (17.8%)	3,722 (33.5%)	474 (33.4%)
60-80%	974,427 (20.0%)	2,391 (21.5%)	302 (21.3%)
80-100%	1,034,469 (21.2%)	2,184 (19.6%)	267 (18.8%)

Abbreviations: SD, Standard Deviation; ICU, Intensive Care Unit

Table description: The exposure groups, *no COVID-19 hospitalisation*, *COVID-19 hospitalisation without ICU*, and *COVID-19 hospitalisation with ICU*, were identified by the end of follow up

[a] The group, *no COVID-19 hospitalisation,* consisted of individuals without admission to a hospital with SARS-CoV-2 infection, i.e., all individuals without PCR test results, with only negative test results, or a positive test result but no admission to a hospital by the end of follow up

[b] The group, *COVID-19 hospitalisation without ICU,* consisted of everyone with at least one admission to a hospital with COVID-19 and without ICU admission by the end of follow up

[c] The group, *COVID-19 hospitalisation with ICU,* consisted of everyone with at least one admission to the hospital with COVID-19 and with ICU admission by the end of follow up

Results: Positive SARS-CoV-2 test

Supplementary Table 3: Risks of any first neurological disorder following a COVID-19 negative test compared to no test across age groups

	Cases, No.			
**Age**	**No SARS-CoV-2 test**	**SARS-CoV-2 negative**	**HR (95% CI)[a]**	**P value**
<20	2,280	4,357	2.08 (1.93 - 2.23)	<0.001
20-29	1,823	4,170	2.06 (1.91 - 2.23)	<0.001
30-39	2,212	4,769	1.84 (1.72 - 1.96)	<0.001
40-49	3,146	6,098	1.72 (1.62 - 1.82)	<0.001
50-59	4,698	8,147	1.64 (1.56 - 1.71)	<0.001
60-69	5,433	7,858	1.72 (1.65 - 1.80)	<0.001
70-79	8,479	9,822	1.73 (1.68 - 1.79)	<0.001
≥80	6,939	6,167	1.75 (1.68 - 1.82)	<0.001

Results are derived from a study population of n = 4,888,615 individuals with 89,013 cases of incident neurological disorders. The HRs with 95% CI and two-sided Wald P values unadjusted for multiple comparisons are from a Cox Proportional Hazards model.
Abbreviations: HR, Hazard Ratio; CI, Confidence Interval

[a] Based on a Cox Proportional Hazards model stratified by age and adjusted for confounders (sex, parental neurology, Charlson Comorbidity Index, employment status, income, highest level of education)

Supplementary Table 4: Risks of any first neurological disorder following a positive SARS-CoV-2 test compared to no SARS-CoV-2 test across age groups

	Cases, No.			
**Age**	**No SARS-CoV-2 test**	**SARS-CoV-2 positive**	**HR (95% CI)[a]**	**P value**
<20	2,280	258	1.85 (1.60 - 2.13)	<0.001
20-29	1,823	321	2.22 (1.94 - 2.54)	<0.001
30-39	2,212	314	2.07 (1.82 - 2.36)	<0.001
40-49	3,146	452	2.21 (1.98 - 2.46)	<0.001
50-59	4,698	456	1.77 (1.60 - 1.96)	<0.001
60-69	5,433	300	1.92 (1.70 - 2.17)	<0.001
70-79	8,479	297	1.97 (1.75 - 2.22)	<0.001
≥80	6,939	217	2.09 (1.82 - 2.40)	<0.001

Results are derived from a study population of n = 4,888,615 individuals with 89,013 cases of incident neurological disorders. The HRs with 95% CI and two-sided Wald P values unadjusted for multiple comparisons are from a Cox Proportional Hazards model.
Abbreviations: HR, Hazard Ratio; CI, Confidence Interval

[a] Based on a Cox Proportional Hazards model stratified by age and adjusted for confounders (sex, parental neurology, Charlson Comorbidity Index, employment status, income, highest level of education)

Supplementary Table 5: Risks of any first neurological disorder following a positive SARS-CoV-2 test compared to a negative SARS-CoV-2 test across age groups

	Cases, No.			
**Age**	**SARS-CoV-2 negative**	**SARS-CoV-2 positive**	**HR (95% CI)[a]**	**P value**
<20	4,357	258	0.89 (0.78 - 1.01)	0.069
20-29	4,170	321	1.08 (0.96 - 1.21)	0.207
30-39	4,769	314	1.13 (1.01 - 1.27)	0.039
40-49	6,098	452	1.29 (1.17 - 1.42)	<0.001
50-59	8,147	456	1.08 (0.98 - 1.19)	0.103
60-69	7,858	300	1.12 (0.99 - 1.25)	0.062
70-79	9,822	297	1.14 (1.01 - 1.28)	0.028
≥80	6,167	217	1.20 (1.04 - 1.37)	0.010

Results are derived from a study population of n = 4,888,615 individuals with 89,013 cases of incident neurological disorders. The HRs with 95% CI and two-sided Wald P values unadjusted for multiple comparisons are from a Cox Proportional Hazards model.

Abbreviations: HR, Hazard Ratio; CI, Confidence Interval

[a] Based on a Cox Proportional Hazards model stratified by age and adjusted for confounders (sex, parental neurology, Charlson Comorbidity Index, employment status, income, highest level of education)

Supplementary Table 6: Risk of specific first neurological outcomes among individuals with negative SARS-CoV-2 test compared to no SARS-CoV-2 test

	Cases, No.			
	**No SARS-CoV-2 test**	**SARS-CoV-2 negative**	**HR (95% CI)[a]**	**P value**
Parkinson	2,396	3,356	1.69 (1.59 - 1.80)	<0.001
Neurodegenerative	3,668	4,040	1.39 (1.31 - 1.46)	<0.001
Dementia	7,609	8,099	1.52 (1.47 - 1.58)	<0.001
Alzheimer’s Disease	3,280	3,172	1.31 (1.24 - 1.39)	<0.001
Vascular Dementia	887	1,216	1.91 (1.73 - 2.11)	<0.001
Immune-mediated	657	1,351	2.13 (1.87 - 2.42)	<0.001
Multiple Sclerosis	525	923	1.54 (1.32 - 1.79)	<0.001
Guillain Barré	36	140	8.95 (5.34 - 15.02)	<0.001
Other Immune-mediated	216	501	2.49 (2.01 - 3.09)	<0.001
Epilepsy	2,037	4,016	3.07 (2.86 - 3.29)	<0.001
Headache	4,703	10,523	2.11 (2.01 - 2.21)	<0.001
Narcolepsy	37	106	7.20 (4.22 - 12.27)	<0.001
Nerve/nerve root & plexus	9,671	15,910	1.56 (1.51 - 1.61)	<0.001
Polyneuropathy	5,207	7,787	1.60 (1.53 - 1.67)	<0.001
Neuromuscular	396	678	1.98 (1.69 - 2.33)	<0.001
Myopathy	148	288	2.23 (1.73 - 2.88)	<0.001
Other Neuromuscular	291	485	2.12 (1.75 - 2.57)	<0.001
Cerebrovascular Disease	12,718	16,671	1.74 (1.69 - 1.79)	<0.001
CNS Infection	445	1,298	5.25 (4.54 - 6.07)	<0.001
Viral CNS Infection	159	479	5.71 (4.47 - 7.28)	<0.001
Other Neurological Disorder	3,728	7,357	2.70 (2.56 - 2.84)	<0.001

Results are derived from a study population of n = 4,888,615 individuals with 89,013 cases of incident neurological disorders. The HRs with 95% CI and two-sided Wald P values unadjusted for multiple comparisons are from a Cox Proportional Hazards model.
Abbreviations: HR, Hazard Ratio; CI, Confidence Interval

[a] Based on a Cox Proportional Hazards model stratified by age and adjusted for confounders (sex, parental neurology, Charlson Comorbidity Index, employment status, income, highest level of education)

Supplementary Table 7: Risks of specific first neurological outcomes among individuals with a positive SARS-CoV-2 test compared to no SARS-CoV-2 test

	Cases, No.			
	No SARS-CoV-2 test	SARS-CoV-2 positive	HR (95% CI)[a]	P value
Parkinson	2,396	142	1.87 (1.57 - 2.23)	<0.001
Neurodegenerative	3,668	204	1.82 (1.58 - 2.11)	<0.001
Dementia	7,609	353	2.13 (1.91 - 2.38)	<0.001
Alzheimer's Disease	3,280	102	1.31 (1.07 - 1.59)	0.009
Vascular Dementia	887	59	2.84 (2.16 - 3.72)	<0.001
Immune-mediated	657	93	2.61 (2.05 - 3.33)	<0.001
Multiple Sclerosis	525	42	1.18 (0.84 - 1.66)	0.330
Guillain Barré	36	10	12.64 (5.60 - 28.50)	<0.001
Other Immune-mediated	216	49	4.67 (3.28 - 6.66)	<0.001
Epilepsy	2,037	160	2.68 (2.26 - 3.18)	<0.001
Headache	4,703	813	2.91 (2.68 - 3.16)	<0.001
Narcolepsy	37	≤5[b]	..	..
Nerve/nerve root & plexus	9,671	719	1.42 (1.31 - 1.54)	<0.001
Polyneuropathy	5,207	277	1.36 (1.20 - 1.54)	<0.001
Neuromuscular	396	114	7.11 (5.58 - 9.07)	<0.001
Myopathy	148	96	16.45 (11.93 - 22.68)	<0.001
Other Neuromuscular	291	40	3.72 (2.58 - 5.35)	<0.001
Cerebrovascular Disease	12,718	681	1.84 (1.70 - 1.99)	<0.001
CNS Infection	445	69	5.81 (4.40 - 7.69)	<0.001
Viral CNS Infection	159	21	5.26 (3.20 - 8.62)	<0.001
Other Neurological Disorder	3,728	403	3.38 (3.02 - 3.77)	<0.001

Results are derived from a study population of n = 4,888,615 individuals with 89,013 cases of incident neurological disorders. The HRs with 95% CI and two-sided Wald P values unadjusted for multiple comparisons are from a Cox Proportional Hazards model.
Abbreviations: HR, Hazard Ratio; CI, Confidence Interval

[a] Based on a Cox Proportional Hazards model stratified by age and adjusted for confounders (sex, parental neurology, Charlson Comorbidity Index, employment status, income, highest level of education)
[b] Results from ≤5 patients were omitted to ensure data privacy

Supplementary Table 8: Risks of specific first neurological outcomes among individuals with a positive SARS-CoV-2 test compared to a negative SARS-CoV-2 test

	Cases, No.			
	SARS-CoV-2 negative	SARS-CoV-2 positive	HR (95% CI)[a]	P value
Parkinson	3,356	142	1.11 (0.93 - 1.31)	0.239
Neurodegenerative	4,040	204	1.32 (1.14 - 1.52)	<0.001
Dementia	8,099	353	1.40 (1.26 - 1.56)	<0.001
Alzheimer's Disease	3,172	102	1.00 (0.82 - 1.21)	0.966
Vascular Dementia	1,216	59	1.49 (1.14 - 1.93)	0.003
Immune-mediated	1,351	93	1.23 (0.99 - 1.52)	0.057
Multiple Sclerosis	923	42	0.77 (0.56 - 1.05)	0.098
Guillain Barré	140	10	1.41 (0.74 - 2.71)	0.300
Other Immune-mediated	501	49	1.88 (1.40 - 2.53)	<0.001
Epilepsy	4,016	160	0.87 (0.74 - 1.02)	0.095
Headache	10,523	813	1.38 (1.28 - 1.48)	<0.001
Narcolepsy	106	≤5[b]	..	..
Nerve/nerve root & plexus	15,910	719	0.91 (0.85 - 0.98)	0.015
Polyneuropathy	7,787	277	0.85 (0.75 - 0.96)	0.007
Neuromuscular	678	114	3.59 (2.93 - 4.39)	<0.001
Myopathy	288	96	7.37 (5.81 - 9.35)	<0.001
Other Neuromuscular	485	40	1.75 (1.27 - 2.43)	0.001
Cerebrovascular Disease	16,671	681	1.06 (0.98 - 1.14)	0.173
CNS Infection	1298	69	1.11 (0.87 - 1.41)	0.414
Viral CNS Infection	479	21	0.92 (0.59 - 1.43)	0.714
Other Neurological Disorder	7,357	403	1.25 (1.13 - 1.39)	<0.001

Results are derived from a study population of n = 4,888,615 individuals with 89,013 cases of incident neurological disorders. The HRs with 95% CI and two-sided Wald P values unadjusted for multiple comparisons are from a Cox Proportional Hazards model.

Abbreviations: HR, Hazard Ratio; CI, Confidence Interval

[a] Based on a Cox Proportional Hazards model stratified by age and adjusted for confounders (sex, parental neurology, Charlson Comorbidity Index, employment status, income, highest level of education)

[b] Results from ≤5 patients were omitted to ensure data privacy

Supplementary Table 9: **Risks of specific first polyneuropathic and neuromuscular outcomes among individuals with a positive SARS-CoV-2 test compared to a negative SARS-CoV-2 test**

ICD-10[c]	Cases, No. SARS-CoV-2 negative	SARS-CoV-2 positive	HR (95% CI)[a]	P value
G60	215	9	0.86 (0.44 – 1.68)	0.652
G60.0-G60.9	..	≤5[b]	..	..
G61	233	15	1.30 (0.76 – 2.20)	0.337
G61.0	140	10	1.41 (0.74 – 2.71)	0.300
G61.1-G61.9	..	≤5[b]	..	..
G62	3,433	123	0.91 (0.76 – 1.09)	0.324
G62.0-G62.2	..	≤5[b]	..	..
G62.8	210	13	1.46 (0.83 – 2.57)	0.188
G62.9	3,208	112	0.89 (0.74 – 1.08)	0.243
G63	1,667	42	0.77 (0.56 – 1.04)	0.090
G63.0-G63.1	..	≤5[b]	..	..
G63.2	1566	36	0.71 (0.51 – 0.99)	0.041
G63.3-G63.8	..	≤5[b]	..	..
G64	152	10	1.39 (0.73 – 2.66)	0.314
G70	214	29	2.96 (2.00 - 4.38)	<0.001
G70.0	167	28	3.69 (2.46 - 5.54)	<0.001
G70.1-9	..	≤5[b]	..	..
G71	225	9	0.79 (0.40 - 1.54)	0.481
G71.0-G71.9	..	≤5[b]	..	..
G72	288	96	7.37 (5.81 - 9.35)	<0.001
G72.0-G72.8		≤5[b]	..	..
G72.9	232	90	8.38 (6.53 - 10.77)	<0.001
G73	..	≤5[b]	..	..

Results are derived from a study population of n = 4,888,615 individuals with 89,013 cases of incident neurological disorders. The HRs with 95% CI and two-sided Wald P values unadjusted for multiple comparisons are from Cox Proportional Hazards models.

Abbreviations: HR, Hazard Ratio; CI, Confidence Interval

[a] Based on a Cox Proportional Hazards model stratified by age and adjusted for confounders (sex, parental neurology, Charlson Comorbidity Index, employment status, income, highest level of education)

[b] Results from ≤5 patients were omitted to ensure data privacy

[c] The ICD-10 codes are used to define both the outcome and exclude individuals with pre-existing neurological disorders.

Supplementary Table 10: Risks of specific first neurological outcomes at different time points since the first SARS-CoV-2 positive test compared with SARS-CoV-2 negative tests

	Months since the first positive test	Cases, No.	HR (95% CI)[a]	P value
Parkinson	SARS-CoV-2 negative	3,356	..	..
	<1	23	1.24 (0.82 - 1.87)	0.315
	1-2	35	1.43 (1.02 - 2.00)	0.035
	3-5	28	0.93 (0.64 - 1.35)	0.689
	6-11	46	1.05 (0.78 - 1.41)	0.739
	12+	10	1.06 (0.57 - 1.98)	0.854
Neurodegenerative	SARS-CoV-2 negative	4,040	..	..
	<1	29	1.26 (0.87 - 1.81)	0.224
	1-2	44	1.44 (1.07 - 1.94)	0.016
	3-5	53	1.43 (1.09 - 1.88)	0.010
	6-11	58	1.12 (0.86 - 1.45)	0.409
	12+	20	1.70 (1.09 - 2.65)	0.018
Dementia	SARS-CoV-2 negative	8,099	..	..
	<1	59	1.53 (1.18 - 1.98)	0.001
	1-2	66	1.32 (1.03 - 1.68)	0.027
	3-5	91	1.58 (1.28 - 1.94)	<0.001
	6-11	112	1.34 (1.11 - 1.62)	0.002
	12+	25	1.22 (0.83 - 1.81)	0.315
- Alzheimer’s Disease	SARS-CoV-2 negative	3,172	..	..
	<1	17	1.19 (0.73 - 1.92)	0.486
	1-2	25	1.26 (0.85 - 1.87)	0.249
	3-5	26	1.13 (0.77 - 1.66)	0.541
	6-11	28	0.77 (0.53 - 1.12)	0.168
	12+	6	0.70 (0.31 - 1.56)	0.383
- Vascular Dementia	SARS-CoV-2 negative	1,216	..	..
	<1	6	1.06 (0.47 - 2.38)	0.883
	1-2	11	1.40 (0.77 - 2.54)	0.271
	3-5	11	1.23 (0.68 - 2.23)	0.496
	6-11	27	2.03 (1.38 - 2.99)	<0.001
	12+	≤5[b]	..	..
Immune-mediated	SARS-CoV-2 negative	1,351	..	..
	<1	17	1.53 (0.95 - 2.48)	0.084
	1-2	12	0.81 (0.46 - 1.43)	0.473
	3-5	26	1.41 (0.95 - 2.08)	0.084
	6-11	29	1.14 (0.79 - 1.66)	0.482
	12+	9	1.70 (0.88 - 3.30)	0.114
- Multiple Sclerosis	SARS-CoV-2 negative	923	..	..
	<1	≤5[b]	..	..
	1-2	6	0.57 (0.26 - 1.28)	0.176
	3-5	15	1.09 (0.65 - 1.82)	0.734
	6-11	12	0.64 (0.36 - 1.13)	0.123
	12+	≤5[b]	..	..
- Guillain Barré	SARS-CoV-2 negative	140	..	..
	<1	6	5.41 (2.31 - 12.65)	<0.001
	1-2	≤5[b]	..	..
	3-5	≤5[b]	..	..
	6-11	≤5[b]	..	..

	Months since the first positive test	Cases, No.	HR (95% CI)[a]	P value
	12+	≤5[b]	..	..
- Other Immune-mediated	SARS-CoV-2 negative	501	..	..
	<1	7	1.87 (0.88 - 3.98)	0.103
	1-2	≤5[b]	..	..
	3-5	13	2.06 (1.18 - 3.58)	0.011
	6-11	19	2.23 (1.40 - 3.55)	0.001
	12+	≤5[b]	..	..
Epilepsy	SARS-CoV-2 negative	4,016	..	..
	<1	23	0.78 (0.52 - 1.18)	0.241
	1-2	41	1.06 (0.78 - 1.45)	0.693
	3-5	35	0.79 (0.57 - 1.11)	0.178
	6-11	46	0.82 (0.61 - 1.10)	0.190
	12+	15	1.31 (0.79 - 2.18)	0.298
Headache	SARS-CoV-2 negative	10,523	..	..
	<1	90	1.04 (0.85 - 1.29)	0.683
	1-2	119	0.97 (0.81 - 1.16)	0.744
	3-5	211	1.40 (1.22 - 1.60)	<0.001
	6-11	325	1.69 (1.51 - 1.89)	<0.001
	12+	68	1.80 (1.42 - 2.29)	<0.001
Narcolepsy	SARS-CoV-2 negative	106	..	..
	<1	≤5[b]	..	..
	1-2	≤5[b]	..	..
	3-5	≤5[b]	..	..
	6-11	≤5[b]	..	..
	12+	≤5[b]	..	..
Nerve/nerve root & plexus	SARS-CoV-2 negative	15,910	..	..
	<1	62	0.57 (0.44 - 0.73)	<0.001
	1-2	149	0.97 (0.82 - 1.14)	0.686
	3-5	201	1.02 (0.89 - 1.17)	0.805
	6-11	255	0.94 (0.83 - 1.07)	0.359
	12+	52	0.89 (0.68 - 1.17)	0.394
Polyneuropathy	SARS-CoV-2 negative	7,787	..	..
	<1	46	0.93 (0.69 - 1.24)	0.619
	1-2	60	0.90 (0.70 - 1.16)	0.417
	3-5	63	0.83 (0.65 - 1.07)	0.150
	6-11	88	0.80 (0.65 - 0.99)	0.044
	12+	20	0.83 (0.53 - 1.29)	0.405
Neuromuscular	SARS-CoV-2 negative	678	..	..
	<1	6	1.23 (0.55 - 2.76)	0.612
	1-2	18	2.59 (1.61 - 4.14)	<0.001
	3-5	33	4.38 (3.07 - 6.26)	<0.001
	6-11	46	4.71 (3.46 - 6.41)	<0.001
	12+	11	5.31 (2.89 - 9.76)	<0.001
- Myopathy	SARS-CoV-2 negative	288	..	..
	<1	≤5[b]	..	..
	1-2	14	4.87 (2.83 - 8.38)	<0.001
	3-5	28	8.98 (6.02 - 13.39)	<0.001
	6-11	41	10.72 (7.58 - 15.15)	<0.001
	12+	11	13.79 (7.34 - 25.88)	<0.001

	Months since the first positive test	Cases, No.	HR (95% CI)[a]	P value
- Other Neuromuscular	SARS-CoV-2 negative	485	..	..
	<1	≤5[b]	..	..
	1-2	≤5[b]	..	..
	3-5	12	2.21 (1.24 - 3.93)	0.007
	6-11	13	1.86 (1.06 - 3.25)	0.030
	12+	6	4.12 (1.82 - 9.37)	0.001
Cerebrovascular Disease	SARS-CoV-2 negative	16,671	..	..
	<1	153	1.58 (1.35 - 1.86)	<0.001
	1-2	128	1.03 (0.87 - 1.23)	0.725
	3-5	143	0.99 (0.84 - 1.17)	0.952
	6-11	211	0.99 (0.86 - 1.13)	0.863
	12+	46	0.92 (0.69 - 1.23)	0.568
CNS Infection	SARS-CoV-2 negative	1,298	..	..
	<1	10	0.92 (0.49 - 1.73)	0.800
	1-2	12	0.97 (0.55 - 1.72)	0.927
	3-5	23	1.62 (1.07 - 2.45)	0.023
	6-11	17	0.84 (0.52 - 1.36)	0.476
	12+	7	1.60 (0.76 - 3.39)	0.218
- Viral CNS Infection	SARS-CoV-2 negative	479	..	..
	<1	≤5[b]	..	..
	1-2	≤5[b]	..	..
	3-5	6	1.12 (0.50 - 2.52)	0.783
	6-11	7	0.94 (0.44 - 1.99)	0.862
	12+	≤5[b]	..	..
Other Neurological Disorder	SARS-CoV-2 negative	7,357	..	..
	<1	54	1.09 (0.83 - 1.43)	0.520
	1-2	83	1.26 (1.01 - 1.57)	0.037
	3-5	110	1.47 (1.21 - 1.77)	<0.001
	6-11	124	1.19 (1.00 - 1.43)	0.053
	12+	32	1.45 (1.02 - 2.05)	0.038

Results are derived from a study population of n = 4,331,649 individuals with 54,003 cases of incident neurological disorders. The HRs with 95% CI and two-sided Wald P values unadjusted for multiple comparisons are from a Cox Proportional Hazards model.
Abbreviations: HR, Hazard Ratio; CI, Confidence Interval

[a] Based on a Cox Proportional Hazards model stratified by age and adjusted for confounders (sex, parental neurology, Charlson Comorbidity Index, employment status, income, highest level of education)
[b] Results from ≤5 patients were omitted to ensure data privacy

Supplementary Figure 1: Plot of the HR and 95% CI for the risks of specific first neurological outcomes at different time points since the first SARS-CoV-2 positive test compared to a negative test.

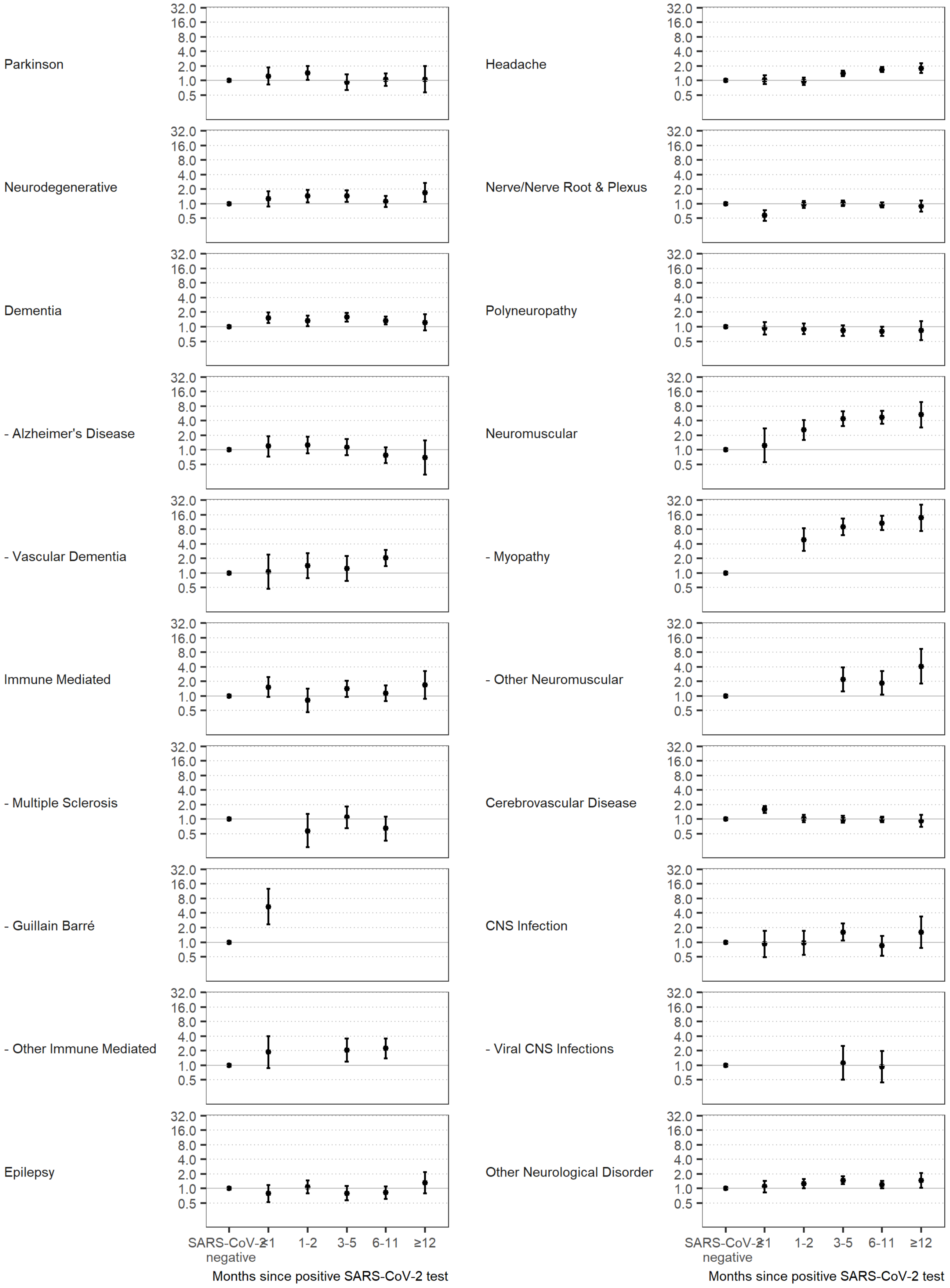

Results are derived from a study population of n = 4,331,649 individuals with a total of 54,003 cases of incident neurological disorders. Estimates are HRs with 95% CI from a Cox Proportional Hazards model stratified by age and adjusted for confounders (sex, parental neurology, Charlson Comorbidity Index, employment status, income, highest level of education). Results from ≤5 patients are omitted to ensure data privacy. See Supplementary Table 18 for ICD-8 and ICD-10 codes included in each neurological disease category. Abbreviations: HR, Hazard Ratio; CI, Confidence Interval. Source data are provided as a Source Data file.

Supplementary Table 11: Risks of specific first neurological outcomes among SARS-CoV-2 positive individuals compared with SARS-CoV-2 negative individuals across age groups

	Age group	Cases, No. SARS-CoV-2 negative	Cases, No. SARS-CoV-2 positive	HR (95% CI)[a]	P value
Parkinson	<20	145	11	1.16 (0.62 - 2.16)	0.638
	20-39	234	15	1.10 (0.65 - 1.86)	0.725
	40-59	665	44	1.26 (0.93 - 1.72)	0.137
	60-79	1,763	51	0.98 (0.74 - 1.29)	0.879
	≥80	549	21	1.14 (0.74 - 1.77)	0.545
Neurodegenerative	<20	85	≤5[b]	..	..
	20-39	509	27	0.82 (0.56 - 1.22)	0.333
	40-59	530	28	0.96 (0.66 - 1.41)	0.843
	60-79	1,687	78	1.59 (1.26 - 1.99)	<0.001
	≥80	1,229	68	1.70 (1.33 - 2.17)	<0.001
Dementia	<20	6	≤5[b]	..	..
	20-39	8	≤5[b]	..	..
	40-59	276	13	0.82 (0.47 - 1.43)	0.487
	60-79	3,989	155	1.34 (1.14 - 1.57)	<0.001
	≥80	3,820	183	1.52 (1.31 - 1.77)	<0.001
- Alzheimer's Disease	<20	≤5[b]	≤5[b]	..	..
	20-39	≤5[b]	≤5[b]	..	..
	40-59	68	≤5[b]	..	..
	60-79	1,500	50	1.14 (0.86 - 1.51)	0.373
	≥80	1,604	50	0.91 (0.69 - 1.21)	0.523
- Vascular Dementia	<20	≤5[b]	≤5[b]	..	..
	20-39	≤5[b]	≤5[b]	..	..
	40-59	28	≤5[b]	..	..
	60-79	606	24	1.35 (0.90 - 2.03)	0.150
	≥80	582	35	1.72 (1.22 - 2.42)	0.002
Immune-mediated	<20	100	6	0.80 (0.35 - 1.84)	0.598
	20-39	550	32	0.89 (0.62 - 1.27)	0.518
	40-59	461	40	1.60 (1.15 - 2.21)	0.005
	60-79	209	13	2.02 (1.15 - 3.54)	0.014
	≥80	31	≤5[b]	..	..
- Multiple Sclerosis	<20	49	≤5[b]	..	..
	20-39	454	20	0.66 (0.42 - 1.03)	0.066
	40-59	340	16	0.88 (0.53 - 1.46)	0.632
	60-79	76	≤5[b]	..	..
	≥80	≤5[b]	≤5[b]	..	..
- Guillain Barré	<20	17	≤5[b]	..	..
	20-39	26	≤5[b]	..	..
	40-59	43	≤5[b]	..	..
	60-79	49	≤5[b]	..	..
	≥80	≤5[b]	≤5[b]	..	..
- Other Immune-mediated	<20	43	≤5[b]	..	..
	20-39	164	16	1.61 (0.96 - 2.71)	0.073
	40-59	168	23	2.50 (1.61 - 3.88)	<0.001
	60-79	104	6	1.78 (0.78 - 4.07)	0.170
	≥80	22	≤5[b]		
	<20	1,025	47	0.75 (0.56 - 1.00)	0.053

	Age group	Cases, No.		HR (95% CI)[a]	P value
		SARS-CoV-2 negative	SARS-CoV-2 positive		
	20-39	484	25	0.82 (0.54 - 1.22)	0.326
Epilepsy	40-59	699	29	0.85 (0.58 - 1.23)	0.380
	60-79	1,351	42	1.06 (0.78 - 1.44)	0.706
	≥80	457	17	1.10 (0.68 - 1.78)	0.705
	<20	2,009	116	0.84 (0.70 - 1.02)	0.079
	20-39	3,791	344	1.45 (1.30 - 1.62)	<0.001
Headache	40-59	3,504	294	1.64 (1.45 - 1.85)	<0.001
	60-79	1,108	56	1.68 (1.28 - 2.19)	<0.001
	≥80	111	≤5[b]	..	..
	<20	22	≤5[b]	..	..
	20-39	52	≤5[b]	..	..
Narcolepsy	40-59	25	≤5[b]	..	..
	60-79	6	≤5[b]	..	..
	≥80	≤5	≤5[b]	..	..
	<20	565	32	0.81 (0.57 - 1.16)	0.250
	20-39	3,398	183	0.84 (0.72 - 0.98)	0.023
Nerve/nerve root & plexus	40-59	6,644	359	1.00 (0.89 - 1.11)	0.928
	60-79	4,563	130	0.90 (0.76 - 1.08)	0.260
	≥80	740	15	0.56 (0.34 - 0.94)	0.028
	<20	241	10	0.65 (0.34 - 1.22)	0.182
	20-39	804	38	0.72 (0.52 - 1.00)	0.050
Polyneuropathy	40-59	2,320	114	0.94 (0.77 - 1.13)	0.487
	60-79	3,738	98	0.87 (0.71 - 1.06)	0.170
	≥80	684	17	0.71 (0.44 - 1.15)	0.164
	<20	70	≤5[b]	..	..
	20-39	127	20	2.53 (1.57 - 4.09)	<0.001
Neuromuscular	40-59	193	65	6.32 (4.75 - 8.42)	<0.001
	60-79	245	21	2.77 (1.77 - 4.34)	<0.001
	≥80	43	≤5[b]	..	..
	<20	20	≤5[b]	..	..
	20-39	51	15	4.90 (2.72 - 8.82)	<0.001
- Myopathy	40-59	96	61	12.11 (8.71 – 16.84)	<0.001
	60-79	103	16	5.33 (3.13 - 9.08)	<0.001
	≥80	18	≤5[b]	..	..
	<20	62	≤5[b]	..	..
	20-39	95	8	1.36 (0.65 - 2.82)	0.412
- Other Neuromuscular	40-59	128	22	3.36 (2.13 - 5.31)	<0.001
	60-79	169	6	1.12 (0.50 - 2.54)	0.781
	≥80	31	≤5[b]	..	..
	<20	103	9	1.29 (0.64 - 2.58)	0.474
	20-39	585	43	1.08 (0.79 - 1.48)	0.623
Cerebrovascular Disease	40-59	3,462	184	0.94 (0.81 - 1.09)	0.378
	60-79	8,806	302	1.09 (0.97 - 1.22)	0.144
	≥80	3,715	143	1.15 (0.97 - 1.35)	0.111
	<20	184	15	1.22 (0.71 - 2.08)	0.470
	20-39	280	23	1.29 (0.84 - 1.98)	0.254
CNS Infection	40-59	309	18	1.11 (0.69 - 1.80)	0.657

	Age group	Cases, No. SARS-CoV-2 negative	SARS-CoV-2 positive	HR (95% CI)[a]	P value
	60-79	417	8	0.68 (0.33 - 1.36)	0.272
	≥80	108	≤5[b]	..	..
- Viral CNS Infection	<20	55	≤5[b]	..	..
	20-39	131	10	1.27 (0.66 - 2.43)	0.475
	40-59	113	≤5[b]	..	..
	60-79	140	≤5[b]	..	..
	≥80	40	≤5[b]	..	..
Other Neurological Disorder	<20	603	39	1.07 (0.77 - 1.48)	0.704
	20-39	1,113	95	1.37 (1.11 - 1.69)	0.004
	40-59	2,051	123	1.16 (0.96 - 1.39)	0.115
	60-79	2,995	118	1.32 (1.10 - 1.59)	0.003
	≥80	595	28	1.41 (0.96 - 2.05)	0.079

Results are derived from a study population of n = 4,888,615 individuals with 89,013 cases of incident neurological disorders. The HRs with 95% CI and two-sided Wald P values unadjusted for multiple comparisons are from Cox Proportional Hazards models.
Abbreviations: HR, Hazard Ratio; CI, Confidence Interval

[a] Based on a Cox Proportional Hazards model stratified by age and adjusted for confounders (sex, parental neurology, Charlson Comorbidity Index, employment status, income, highest level of education)
[b] Results from ≤5 patients were omitted to ensure data privacy

Supplementary Figure 2: Forest plot of the association between SoV-2 positive test result and specific neurological outcomes across age groups compared to SARS-CoV-2 negative test result

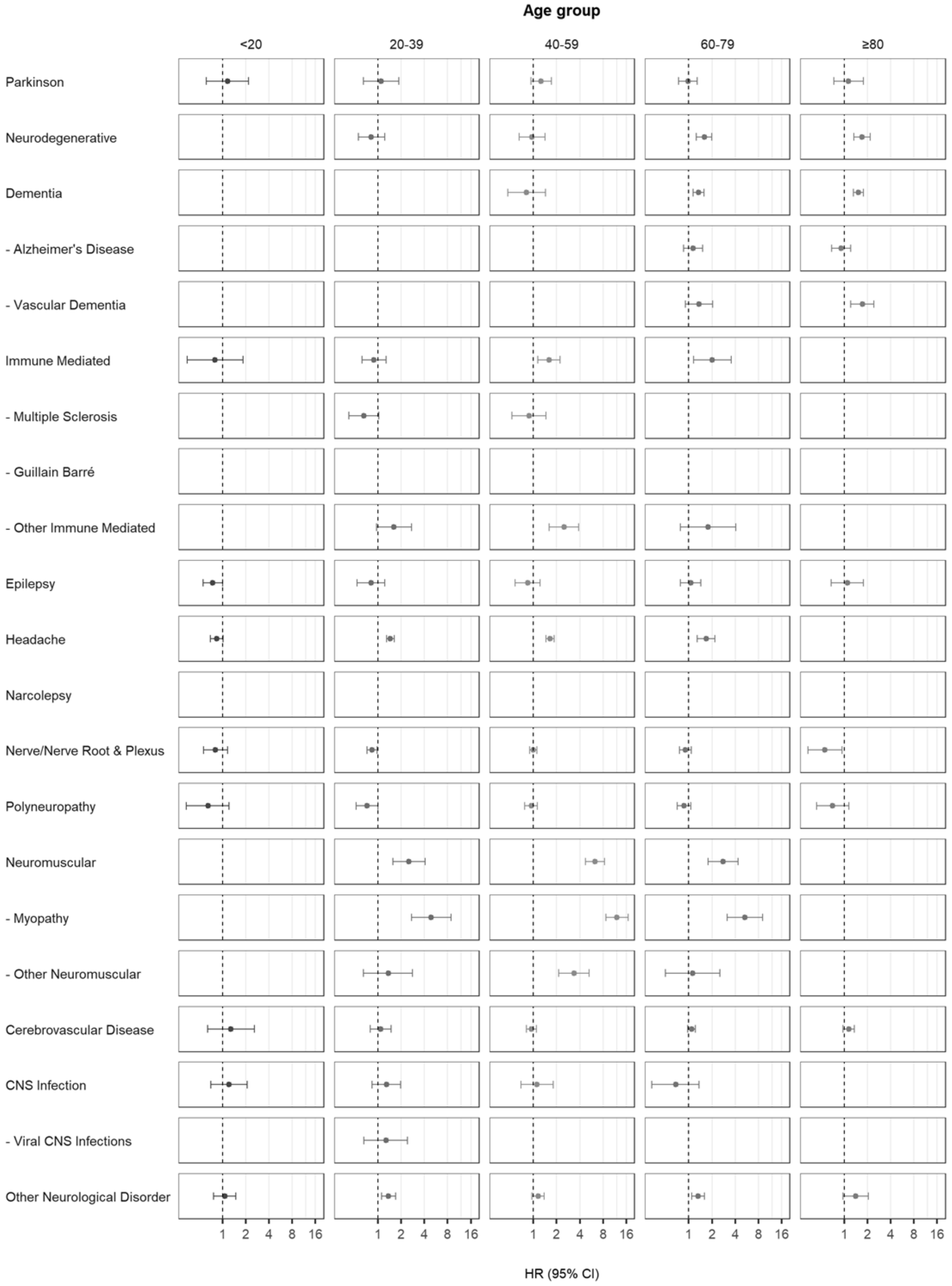

Results are derived from a study population of n = 4,888,615 individuals with a total of 89,013 cases. Estimates are HRs with 95% CI from a Cox Proportional Hazards model stratified by age and adjusted for confounders (sex, parental neurology, Charlson Comorbidity Index, employment status, income, highest level of education). Results from ≤5 patients were omitted to ensure data privacy. See Supplementary Table 18 for ICD-8 and ICD-10 codes included in each neurological disease category. Abbreviations: HR, Hazard Ratio; CI, Confidence Interval. Source data are provided as a Source Data file.

Results: Hospitalisation

Supplementary Table 12: Risks of any first neurological disorder following COVID-19-related hospitalisation with and without ICU admission compared to no COVID-19-related hospitalisation across age groups

	No COVID-19-admission[c]	COVID-19-admission without ICU admission			COVID-19-admission with ICU admission		
**Age**	**Cases, No.**	**Cases, No.**	**HR (95% CI)[a]**	**P value**	**Cases, No.**	**HR (95% CI)[a]**	**P value**
<20	68,89	6	5.65 (2.54 - 12.58)	<0.001	≤5[b]	..	..
20-39	13,578	24	2.48 (1.66 - 3.70)	<0.001	7	13.61 (6.49 - 28.56)	<0.001
40-59	22,899	74	2.69 (2.14 - 3.38)	<0.001	24	8.01 (5.37 - 11.96)	<0.001
60-79	31,987	159	2.86 (2.44 - 3.34)	<0.001	43	4.83 (3.58 - 6.52)	<0.001
≥80	13,224	96	1.97 (1.61 - 2.41)	<0.001	≤5[b]	..	..

Results are derived from a study population of n = 4,888,615 individuals with 89,013 cases of incident neurological disorders. The HRs with 95% CI and two-sided Wald P values unadjusted for multiple comparisons are from Cox Proportional Hazards models.

Abbreviations: HR, Hazard Ratio; CI, Confidence Interval; ICU, Intensive Care Unit

[a] Based on a Cox Proportional Hazards model stratified by age and adjusted for confounders (sex, parental neurology, Charlson Comorbidity Index, employment status, income, highest level of education)

[b] Results from ≤5 patients were omitted to ensure data privacy

[c] The reference group, no COVID-19-admission, consisted of individuals without admission to a hospital with SARS-CoV-2 infection, i.e., all individuals without a PCR test result, with a negative test result, or positive test results but no admission to a hospital

Supplementary Table 13: Risks of specific neurological disorders for COVID-19-related hospitalisation with and without ICU admission compared to individuals without admission to a hospital with SARS-CoV-2 infection

	No COVID-19 admission[c]	COVID-19-hospitalised patients without ICU			COVID-19-hospitalised patients in ICU		
	Cases, No.	Cases, No.	HR (95% CI)[a]	P value	Cases, No.	HR (95% CI)[a]	P value
Parkinson	5,865	25	1.82 (1.23 - 2.70)	0.003	≤5[b]	..	..
Neurodegenerative	7,850	58	2.79 (2.15 - 3.62)	<0.001	≤5[b]	..	..
Dementia	15,925	131	2.50 (2.11 - 2.98)	<0.001	≤5[b]	..	..
Alzheimer's Disease	6,520	32	1.39 (0.98 - 1.97)	0.061	≤5[b]	..	..
Vascular Dementia	2,139	22	2.49 (1.64 - 3.80)	<0.001	≤5[b]	..	..
Immune-mediated	2,089	10	4.06 (2.18 - 7.57)	<0.001	≤5[b]	..	..
Multiple Sclerosis	1,487	≤5[b]	..	..	≤5[b]	..	..
Guillain Barré	181	≤5[b]	..	..	≤5[b]	..	..
Other Immune-mediated	762	≤5[b]	..	..	≤5[b]	..	..
Epilepsy	6,172	34	3.08 (2.20 - 4.32)	<0.001	7	6.13 (2.92 - 12.87)	<0.001
Headache	15,990	46	2.91 (2.18 - 3.90)	<0.001	≤5[b]	..	..
Narcolepsy	148	≤5[b]	..	..	≤5[b]	..	..
Nerve/nerve root & plexus disorders	26,222	58	1.39 (1.08 - 1.80)	0.012	20	4.55 (2.93 - 7.05)	<0.001
Polyneuropathy	13,208	44	1.41 (1.05 - 1.90)	0.023	19	5.44 (3.47 - 8.53)	<0.001
Neuromuscular	1,155	20	10.71 (6.85 - 16.74)	<0.001	13	61.34 (35.35 - 106.42)	<0.001
Myopathy	503	17	20.03 (12.24 - 32.76)	<0.001	12	125.39 (70.11 - 224.24)	<0.001
Other Neuromuscular	811	≤5[b]	..	..	≤5[b]	..	..
Cerebrovascular Disease	29,869	168	2.19 (1.88 - 2.55)	<0.001	33	4.31 (3.06 - 6.06)	<0.001
CNS Infection	1,798	12	3.93 (2.22 - 6.95)	<0.001	≤5[b]	..	..
Viral CNS Infection	655	≤5[b]	..	..	≤5[b]	..	..
Other Neurological Disorder	11,385	88	4.14 (3.35 - 5.11)	<0.001	15	6.27 (3.78 - 10.41)	<0.001

Results are derived from a study population of n = 4,888,615 individuals with 89,013 cases of incident neurological disorders. The HRs with 95% CI and two-sided Wald P values unadjusted for multiple comparisons are from Cox Proportional Hazards models.

Abbreviations: HR, Hazard Ratio; CI, Confidence Interval; ICU, Intensive Care Unit

[a] Based on a Cox Proportional Hazards model stratified by age and adjusted for confounders (sex, parental neurology, Charlson Comorbidity Index, employment status, income, highest level of education)

[b] Results from ≤5 patients were omitted to ensure data privacy

[c] The reference group, no COVID-19-admission, consisted of individuals without admission to a hospital with SARS-CoV-2 infection, i.e., all individuals without a PCR test result, with a negative test result, or positive test results but no admission to a hospital

Supplementary Table 14: Specific neurological disorders for COVID-19-related hospitalisations without admission to ICU compared to individuals without admission to a hospital with COVID-19 across age groups

		Cases, No.		Admission (without ICU) vs. no admission	
	Age group	No admission[c]	Admission (without ICU)	HR (95% CI)[a]	P value
	<20	227	≤5[b]	..	..
	20-39	349	≤5[b]	..	..
Parkinson	40-59	1,059	≤5[b]	..	..
	60-79	3,176	12	1.70 (1.96 - 2.99)	0.068
	≥80	1,054	8	1.65 (0.82 - 3.30)	0.161
	<20	110	≤5[b]	..	..
	20-39	759	≤5[b]	..	..
Neurodegenerative	40-59	870	≤5[b]	..	..
	60-79	3,408	27	3.66 (2.50 - 5.35)	<0.001
	≥80	2,703	28	2.43 (1.67 - 3.53)	<0.001
	<20	11	≤5[b]	..	..
	20-39	13	≤5[b]	..	..
Dementia	40-59	489	≤5[b]	..	..
	60-79	7,544	56	1.60 (1.08 - 2.37)	0.018
	≥80	7,868	73	1.26 (0.93 - 1.71)	0.137
	<20	≤5[b]	≤5[b]	..	..
	20-39	≤5[b]	≤5[b]	..	..
- Alzheimer's Disease	40-59	135	≤5[b]	..	..
	60-79	2,980	8	1.10 (0.55 - 2.20)	0.792
	≥80	3,405	23	1.49 (0.99 - 2.25)	0.057
	<20	≤5[b]	≤5[b]	..	..
	20-39	≤5[b]	≤5[b]	..	..
- Vascular Dementia	40-59	56	≤5[b]	..	..
	60-79	1,002	8	2.66 (1.33 - 5.35)	0.006
	≥80	1,080	14	2.46 (1.45 - 4.17)	0.001
	<20	131	≤5[b]	..	..
	20-39	813	≤5[b]	..	..
Immune-mediated	40-59	742	≤5[b]	..	..
	60-79	357	6	9.07 (4.03 - 20.41)	<0.001
	≥80	46	≤5[b]	..	..
	<20	64	≤5[b]	..	..
	20-39	690	≤5[b]	..	..
- Multiple Sclerosis	40-59	567	≤5[b]	..	..
	60-79	161	≤5[b]	..	..
	≥80	≤5[b]	≤5[b]	..	..
	<20	24	≤5[b]	..	..
	20-39	33	≤5[b]	..	..
- Guillain Barré	40-59	53	≤5[b]	..	..
	60-79	63	≤5[b]	..	..
	≥80	8	≤5[b]	..	..
	<20	54	≤5[b]	..	..
	20-39	222	≤5[b]	..	..
- Other Immune-mediated	40-59	275	≤5[b]	..	..
	60-79	178	≤5[b]	..	..
	≥80	33	≤5[b]	..	..
	<20	1,699	≤5[b]	..	..
	20-39	753	≤5[b]	..	..

		Cases, No.		Admission (without ICU) vs. no admission	
	Age group	No admission[c]	Admission (without ICU)	HR (95% CI)[a]	P value
Epilepsy	40-59	1,040	≤5[b]	..	..
	60-79	1,966	17	3.29 (2.04 - 5.31)	<0.001
	≥80	714	7	2.03 (0.96 - 4.27)	0.064
Headache	<20	3,047	≤5[b]	..	..
	20-39	5,700	9	1.93 (1.00 - 3.72)	0.048
	40-59	5,253	22	3.51 (2.31 - 5.33)	<0.001
	60-79	1,780	12	3.54 (2.00 - 6.25)	<0.001
	≥80	210	≤5[b]	..	..
Narcolepsy	<20	34	≤5[b]	..	..
	20-39	70	≤5[b]	..	..
	40-59	31	≤5[b]	..	..
	60-79	11	≤5[b]	..	..
	≥80	≤5[b]	≤5[b]	..	..
Nerve/nerve root & plexus	<20	846	≤5[b]	..	..
	20-39	5,240	8	1.92 (0.96 - 3.84)	0.066
	40-59	10,620	26	1.93 (1.31 - 2.83)	0.001
	60-79	7,938	17	1.03 (0.64 - 1.67)	0.888
	≥80	1,578	7	0.95 (0.45 - 1.99)	0.883
Polyneuropathy	<20	379	≤5[b]	..	..
	20-39	1,170	≤5[b]	..	..
	40-59	3,800	13	2.17 (1.26 - 3.74)	0.005
	60-79	6,494	22	1.28 (0.84 - 1.94)	0.255
	≥80	1,365	7	1.01 (0.48 - 2.13)	0.978
Neuromuscular	<20	141	≤5[b]	..	..
	20-39	197	≤5[b]	..	..
	40-59	345	9	17.67 (9.08 - 34.40)	<0.001
	60-79	401	8	9.09 (4.50 - 18.39)	<0.001
	≥80	71	≤5[b]	..	..
- Myopathy	<20	46	≤5[b]	..	..
	20-39	85	≤5[b]	..	..
	40-59	188	10	33.18 (17.43 - 63.16)	<0.001
	60-79	156	6	17.44 (7.65 - 39.77)	<0.001
	≥80	28	≤5[b]	..	..
- Other Neuromuscular	<20	116	≤5[b]	..	..
	20-39	143	≤5[b]	..	..
	40-59	217	≤5[b]	..	..
	60-79	285	≤5[b]	..	..
	≥80	50	≤5[b]	..	..
Cerebrovascular Disease	<20	165	≤5[b]	..	..
	20-39	879	≤5[b]	..	..
	40-59	5,624	16	1.92 (1.17 - 3.14)	0.009
	60-79	15,709	89	2.58 (2.10 - 3.18)	<0.001
	≥80	7,492	59	1.78 (1.38 - 2.30)	<0.001
CNS Infection	<20	274	≤5[b]	..	..
	20-39	388	≤5[b]	..	..
	40-59	428	≤5[b]	..	..
	60-79	529	≤5[b]	..	..
	≥80	179	≤5[b]	..	..
	<20	75	≤5[b]	..	..

		Cases, No.		Admission (without ICU) vs. no admission	
	Age group	No admission[c]	Admission (without ICU)	HR (95% CI)[a]	P value
- Viral CNS Infection	20-39	180	≤5[b]	..	..
	40-59	145	≤5[b]	..	..
	60-79	185	≤5[b]	..	..
	≥80	70	≤5[b]	..	..
Other Neurological Disorder	<20	1,057	≤5[b]	..	..
	20-39	1,662	≤5[b]	..	..
	40-59	3,022	22	5.05 (3.22 - 7.69)	<0.001
	60-79	4,618	44	4.04 (3.00 - 5.44)	<0.001
	≥80	1,026	17	3.67 (2.27 - 5.94)	<0.001

Results are derived from a study population of n = 4,888,615 individuals with 89,013 cases of incident neurological disorders. The HRs with 95% CI and two-sided Wald P values unadjusted for multiple comparisons are from a Cox Proportional Hazards model.
Abbreviations: HR, Hazard Ratio; CI, Confidence Interval; ICU, Intensive Care Unit

[a] Based on a Cox Proportional Hazards model stratified by age and adjusted for confounders (sex, parental neurology, Charlson Comorbidity Index, employment status, income, highest level of education)
[b] Results from ≤5 patients were omitted to ensure data privacy
[c] The reference group, no COVID-19-admission, consisted of individuals without admission to a hospital with SARS-CoV-2 infection, i.e., all individuals without a PCR test result, with a negative test result, or positive test results but no admission to a hospital

Supplementary Figure 3: Forest plot of the risks associated with admissions to hospital with COVID-19 and specific neurological outcome across age groups compared to individuals without hospital admission with COVID-19

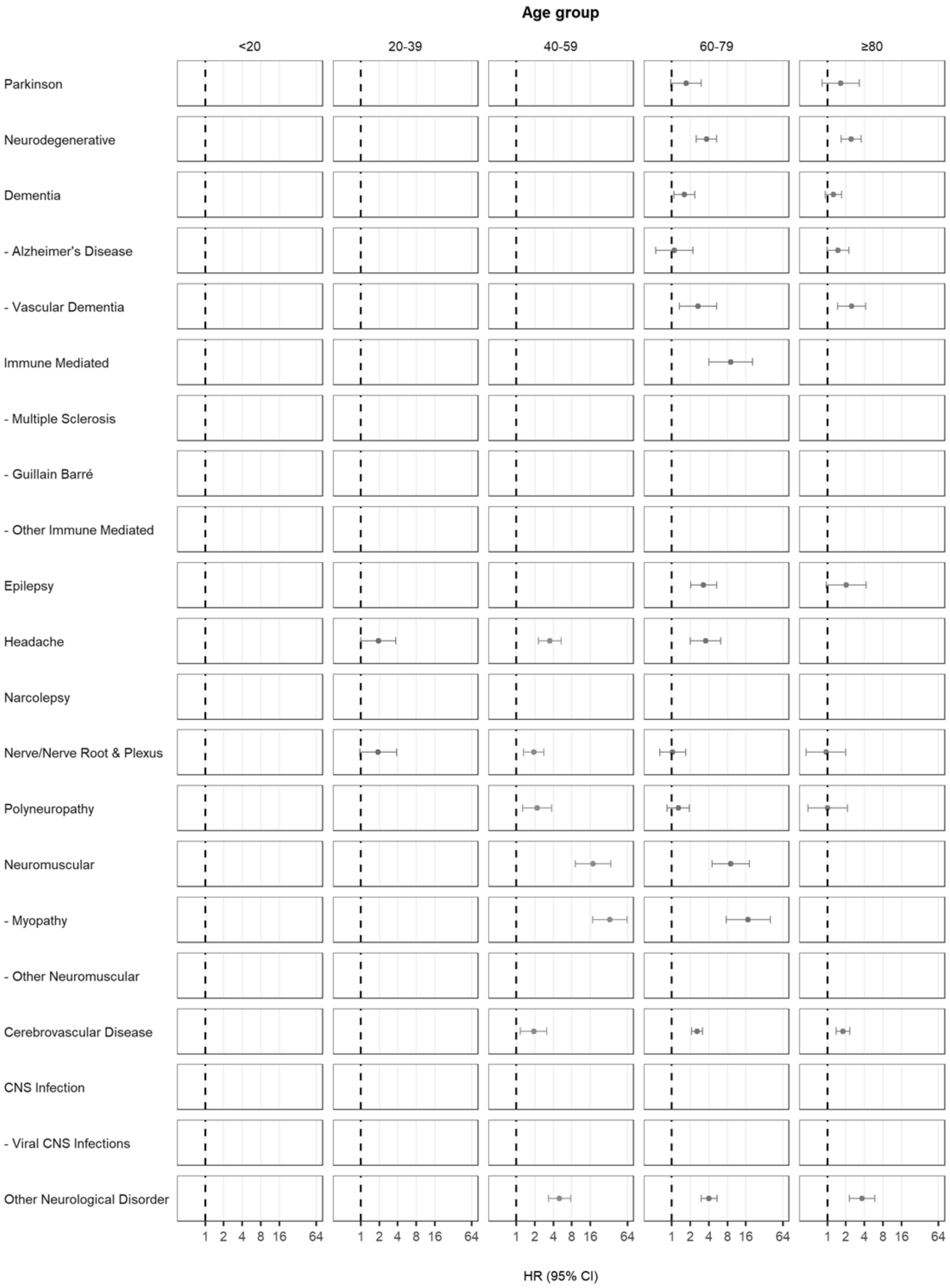

Results are derived from a study population of n = 4,888,615 individuals with a total of 89,013 cases. Estimates are HRs with 95% CI from a Cox Proportional Hazards model stratified by age and adjusted for confounders (sex, parental neurology, Charlson Comorbidity Index, employment status, income, highest level of education). Results from ≤5 patients were omitted to ensure data privacy. The reference group, no COVID-19-admission, consisted of individuals without admission to a hospital with SARS-CoV-2 infection, i.e., all individuals without PCR test results, with negative test results, or positive test results but no admission to a hospital. See Supplementary Table 18 for ICD-8 and ICD-10 codes included in each neurological disease category. Abbreviations: HR, Hazard Ratio; CI, Confidence Interval. Source data are provided as a Source Data file.

Supplementary Table 15: Specific neurological disorders for hospitalised individuals with COVID-19 with ICU admission compared to individuals without COVID-19-admission across age groups

		Cases, No.		ICU admission vs. no admission	
	**Age group**	**No admission**	**ICU admission**	**HR (95% CI)[a]**	**P value**
Nerve/nerve root & plexus	<20	846	≤5[b]	..	..
	20-39	5,240	≤5[b]	..	..
	40-59	10,620	11	7.07 (3.91 - 12.77)	<0.001
	60-79	7,938	7	2.90 (1.38 - 6.09)	0.005
	≥80	1,578	≤5[b]	..	..
Polyneuropathy	<20	379	≤5[b]	..	..
	20-39	1,170	≤5[b]	..	..
	40-59	3,800	≤5[b]	..	..
	60-79	6,494	13	5.28 (3.06 - 9.10)	<0.001
	≥80	1,365	≤5[b]	..	..
Neuromuscular	<20	141	≤5[b]	..	..
	20-39	197	≤5[b]	..	..
	40-59	345	≤5[b]	..	..
	60-79	401	8	60.72 (30.06 - 122.64)	<0.001
	≥80	71	≤5[b]	..	..
- Myopathy	<20	46	≤5[b]	..	..
	20-39	85	≤5[b]	..	..
	40-59	188	≤5[b]	..	..
	60-79	156	7	137.03 (63.82 - 294.21)	<0.001
	≥80	28	≤5[b]	..	..
Cerebrovascular Disease	<20	165	≤5[b]	..	..
	20-39	879	≤5[b]	..	..
	40-59	5,624	11	10.09 (5.58 - 18.24)	<0.001
	60-79	15,709	19	3.39 (2.16 - 5.32)	<0.001
	≥80	7,492	≤5[b]	..	..
Other Neurological Disorder	<20	1,057	≤5[b]	..	..
	20-39	1,662	≤5[b]	..	..
	40-59	3,022	≤5[b]	..	..
	60-79	4,618	9	5.51 (2.86 - 10.60)	<0.001
	≥80	1,026	≤5[b]	..	..

Results are derived from a study population of n = 4,888,615 individuals with 89,013 cases of incident neurological disorders. The HRs with 95% CI and two-sided Wald P values unadjusted for multiple comparisons are from a Cox Proportional Hazards model. Outcome categories with ≤5 patients omitted from the table.
Abbreviations: HR, Hazard Ratio; CI, Confidence Interval; ICU, Intensive Care Unit

[a] Based on a Cox Proportional Hazards model stratified by age and adjusted for confounders (sex, parental neurology, Charlson Comorbidity Index, employment status, income, highest level of education)
[b] Results from ≤5 patients were omitted to ensure data privacy
[c] The reference group, no COVID-19-admission, consisted of individuals without admission to a hospital with SARS-CoV-2 infection, i.e., all individuals without a PCR test result, with a negative test result, or positive test results but no admission to a hospital

Supplementary Table 16: Number of individuals with other infections

	Individuals, No.
**Prescription for anti-infective agent**[a]	
No infection[b]	3,652,879
Any prescription for anti-infective agents	816,126
Antibacterial	746,107
Antivirals	39,870
Antimycotics	30,149
SARS-CoV-2 positive	499,948
**Hospitalisation for pulmonary infection**[c]	
No admission[d]	4,685,103
Any non-COVID pulmonary infection in a hospital	20,171
Influenza	253
Bacterial pneumonia	15,685
Other pulmonary infection	4,232
COVID-19-hospitalisation	11,242

[a] Excluding individuals with a redeemed prescription for any anti-infective agent within the past year to rule out recurring infections.

[b] Individuals without SARS-CoV-2 positive result or prescription for anti-infective agents within the given period

[c] Excluding individuals with any pulmonary infection in the hospital within the past 10 years to rule out recurring infections

[d] Individuals without hospital contact for any pulmonary infection or COVID-19 within the given period, i.e., all individuals not admitted to a hospital with pulmonary infection with either no test result or negative test result, and individuals with positive test results but no admission to a hospital

Supplementary Table 17: Specificity of risks associated with SARS-CoV-2 compared to any prescription for anti-infective agents, and of COVID-19 hospitalisation compared to non-COVID-19 lung infection treated in hospital

		Infection type vs. no infection		COVID-19 vs. infection type	
	**Cases, No.**	**HR (95% CI)[a]**	**P value**	**HR (95% CI)[a]**	**P value**
**Prescription for anti-infective agent[b]**					
No infection[c]	44,529	1.00 [reference]	..	..	..
Any prescription for anti-infective agents	11,539	1.57 (1.54 - 1.61)	<0.001	0.84 (0.80 - 0.89)	<0.001
Antibacterial	10,548	1.57 (1.53 - 1.60)	<0.001	0.85 (0.80 - 0.89)	<0.001
Antivirals	540	1.49 (1.36 - 1.62)	<0.001	0.89 (0.81 - 0.98)	0.022
Antimycotics	451	1.82 (1.66 - 2.00)	<0.001	0.73 (0.65 - 0.81)	<0.001
SARS-CoV-2 positive	1,606	1.33 (1.26 - 1.39)	<0.001	..	..
**Hospitalisation for lung infection[d]**					
No admission[e]	82,210	1.00 [reference]	..	..	..
Any non-COVID pulmonary infection	824	2.72 (2.54 - 2.92)	<0.001	1.06 (0.94 - 1.20)	0.328
Influenza	17	2.47 (1.54 - 3.97)	<0.001	1.18 (0.73 - 1.92)	0.498
Bacterial pneumonia	722	2.71 (2.51 - 2.91)	<0.001	1.08 (0.95 - 1.22)	0.224
Other	85	3.40 (2.75 - 4.21)	<0.001	0.86 (0.68 - 1.09)	0.203
COVID-19-hospitalisation	380	2.92 (2.64 - 3.23)	<0.001	..	..

Results are derived from a study population of n = 4,888,615 individuals with 89,013 cases of incident neurological disorders. The HRs with 95% CI and two-sided Wald P values unadjusted for multiple comparisons are from Cox Proportional Hazards models.

Abbreviations: HR, Hazard Ratio; CI, Confidence Interval

The number of individuals in each category is shown in Supplementary Table 22.

[a] Based on a Cox Proportional Hazards model stratified by age and adjusted for confounders (sex, parental neurology, Charlson Comorbidity Index, occupational status, income, and highest level of education)

[b] Excluding individuals with a redeemed prescription for any anti-infective agent within the past year to rule out recurring infections

[c] The reference group, no infection, consisted of all individuals without a prescription for an anti-infective agent and negative or no test result

[d] Excluding individuals with any lung infection in a hospital within the past 10 years to rule out recurring infections. pulmonary

[e] The reference group, no admission, consisted of all individuals without admission to a hospital with SARS-CoV-2 infection or any pulmonary infection, i.e., all individuals not admitted to a hospital with pulmonary infection with either no test result or negative test result, and individuals with positive test results but no admission to a hospital

Sensitivity Analysis

Sensitivity analysis 1: Various levels of adjusting for confounders

Results from the primary outcome analysis were tested by additionally adjusting for pre-existing comorbidities that might increase the risk of having a severe infection with SARS-CoV-2 or developing neurological disorders (heart disease, autoimmune disease, and head trauma or fractures within 2 years) (Supplementary Table 3) and parents' highest educational level. Overall, the results did not change.

**Adjustment**				**HR (95% CI)**	**P value**
	Negative test	vs.	No test	1.32 (1.29 - 1.34)	<0.001
**Unadjusted**[a]	Positive test	vs.	No test	1.14 (1.10 - 1.19)	<0.001
	Positive test	vs.	Negative test	0.87 (0.83 - 0.90)	<0.001
	Negative test	vs.	No test	1.78 (1.75 - 1.81)	<0.001
**Semi adjusted**[b]	Positive test	vs.	No test	1.96 (1.88 - 2.04)	<0.001
	Positive test	vs.	Negative test	1.10 (1.06 - 1.15)	<0.001
	Negative test	vs.	No test	1.76 (1.73 - 1.79)	<0.001
**Adjusted**[c]	Positive test	vs.	No test	1.96 (1.88 - 2.05)	<0.001
	Positive test	vs.	Negative test	1.11 (1.07 - 1.16)	<0.001
	Negative test	vs.	No test	1.74 (1.71 - 1.77)	<0.001
**Fully adjusted**[d]	Positive test	vs.	No test	1.94 (1.86 - 2.02)	<0.001
	Positive test	vs.	Negative test	1.11 (1.07 - 1.16)	<0.001

All models have calendar time as the underlying timescale and stratification by age in 10-year bands.
Abbreviations: HR, Hazard Ratio; CI, Confidence Interval

[a] Unadjusted model has no confounders adjustment
[b] Semi adjusted model is only adjusted for sex
[c] Adjusted for sex, parental neurology, Charlson Comorbidity Index, employment status, income, the highest level of education
[d] Adjusted for sex, parental neurology, Charlson Comorbidity Index, employment status, income, the highest level of education, other comorbidities (trauma within 2 years, heart disease, and autoimmune disease), and parents' highest attained level of education

Sensitivity analysis 2: Immigration status

The purpose of the analysis was to investigate how immigrants and their descendants influence results because information on pre-existing comorbidities and parental information is not available before immigration. Based on immigration status, all individuals were divided into three groups, i) born in Denmark, ii) immigrated to Denmark, and iii) descendants of immigrants. Positive SARS-CoV-2 PCR tests were compared with negative SARS-CoV-2 PCR tests within each group. The effect modification with immigration status was not significant (P value 0.451), which meant that the test result alone explains the risk of neurological disorders and that immigration status did not influence the outcome.

	**Cases, No.**				
	**Negative SARS-CoV-2**	**Positive SARS-CoV-2**	**HR (95% CI)**[a]	**P value**	**P value of effect modifier**
Born in Denmark	46,329	2,020	1.12 (1.07 - 1.17)	<0.001	0.451
Immigrants	839	144	1.09 (0.91 - 1.30)	0.356	0.451
Immigrant descendants	4,220	451	1.05 (0.95 - 1.15)	0.351	0.451

Abbreviations: HR, Hazard Ratio; CI, Confidence Interval

[a] Based on a Cox Proportional Hazards model stratified by age and adjusted for confounders (sex, parental neurology, Charlson Comorbidity Index, employment status, income, highest level of education)

Sensitivity analysis 3: Prescription for a pre-existing neurological disorder

We also investigated how results depended on individuals with pre-existing neurological disorders treated with medication. Individuals were divided into two groups based on whether they had redeemed a prescription for a neurological disorder before the start of follow up- (ATC codes: N03, N04, N06DX01, N06DA02, N06DA03, N06DA04, N02C) (Supplementary Table 1). The effect of including this variable as an interaction with the PCR test result was significant (P value <0.001), but effects were still increased in both groups. The effect was highest for individuals who had redeemed a prescription for any neurological disorder before the start of follow-up.

	**Cases, No.**				
	**Negative SARS-CoV-2**	**Positive SARS-CoV-2**	**HR (95% CI)[a]**	**P value**	**P value of effect modifier**
No prescription	38,251	1,959	1.06 (1.01 - 1.11)	0.013	<0.001
Prescription	13,137	656	1.27 (1.18 - 1.38)	<0.001	<0.001

Abbreviations: HR, Hazard Ratio; CI, Confidence Interval

[a] Based on a Cox Proportional Hazards model stratified by age and adjusted for confounders (sex, parental neurology, Charlson Comorbidity Index, employment status, income, highest level of education)

Sensitivity analysis 4: Number of SARS-CoV-2 PCR tests

We explored how peoples' testing behaviour affected results by adjusting for the number of SARS-CoV-2 PCR tests conducted per individual by effect modification. People were divided into four groups: 0-3 tests, 4-9 tests, 10-14 tests, and 15 or more tests. Afterwards, the results for positive test results were compared to negative test results within each group. The effect of the number of tests was significant (P value <0.001), but the risk of neurological disorders remained increased for individuals with 4-9 and 10-14 tests The effect was no longer significant for individuals with 0-3 and more than 15 tests.

	**Cases, No.**				
	**Negative SARS-CoV-2**	**Positive SARS-CoV-2**	**HR (95% CI)[a]**	**P value**	**P value of effect modifier**
0-3 tests	30,165	829	0.96 (0.89 - 1.03)	0.226	<0.001
4-9 tests	13,667	1,123	1.11 (1.05 - 1.18)	0.001	<0.001
10-14 tests	3,570	377	1.35 (1.21 - 1.50)	<0.001	<0.001
≥15 tests	3,986	286	1.12 (0.99 - 1.26)	0.063	<0.001

Abbreviations: HR, Hazard Ratio; CI, Confidence Interval

[a] Based on a Cox Proportional Hazards model stratified by age and adjusted for confounders (sex, parental neurology, Charlson Comorbidity Index, employment status, income, and highest level of education)

Sensitivity analysis 5: Effect of lockdown

The effect of lockdowns was analysed by dividing the underlying calendar time into five groups defined in the table below.[1]

Calendar period	Date from	Date to
1st lockdown	2020-03-01	2020-04-16
1st inter lockdown	2020-04-17	2020-12-20
2nd lockdown	2020-12-21	2021-04-21
2nd inter lockdown	2021-04-22	2021-12-18
3rd lock-down	2021-12-19	2022-01-12

The effect of lockdown was assessed by including it as an effect modifier and stratifying by it. Positive SARS-CoV-2 test was compared with negative SARS-CoV-2 tests within each lockdown period. The effect modifier was significant (P value 0.019), but effects within each group were still increased or non-significant. Analysis should be interpreted with caution since the age groups dominating the exposure groups at different points in calendar time varies. Therefore, it is equally likely that the underlying effect was due to differences in age groups and not calendar time.

	Cases, No.				
	Negative SARS-CoV-2	Positive SARS-CoV-2	HR (95% CI)[a]	P value	P value of effect modifier
1st lockdown	113	20	1.61 (1.00 - 2.60)	0.449	0.019
1st inter lockdown	14,035	243	1.05 (0.93 - 1.19)	0.449	0.019
2nd lockdown	12,000	648	1.23 (1.13 - 1.33)	<0.001	0.019
2nd inter lockdown	24,311	1,603	1.10 (1.04 - 1.15)	<0.001	0.019
3rd lock-down	929	101	0.94 (0.77 - 1.16)	0.575	0.019

Abbreviations: HR, Hazard Ratio; CI, Confidence Interval

[a] Based on a Cox Proportional Hazards model stratified by age and adjusted for confounders (sex, parental neurology, Charlson Comorbidity Index, employment status, income, and highest level of education)

Sensitivity analysis 6: Effect of virus variants

Different strains of SARS-CoV-2 have been connected to different levels of contagiousness and severity. The time periods where the variants were most dominant in Denmark are defined in the table below.[1]

Dominating virus variant	Date from	Date to
Wild type	2020-03-01	2021-02-12
Alpha (B.1.1.7)	2021-02-13	2021-06-27
Delta (B.1.617.2)	2021-06-28	2021-12-18
Omicron (B.1.1.529)	2021-12-19	2021-12-31

To explore whether different virus variants have exposed individuals to different levels of risk, we included it as an effect modifier and stratified by it. The overall effect was significant (P value 0.027), but risks were in general still increased except for the Omicron variant, which was not associated with a significantly increased risk. Analysis should be interpreted with caution since the age groups dominating the exposure groups at different points in calendar time varies. Therefore, it is equally likely that the underlying effect was due to differences in age groups and not dominating virus variants.

	Cases, No.				
	Negative SARS-CoV-2	Positive SARS-CoV-2	HR (95% CI)[a]	P value	P value of effect modifier
Wild type	19,072	516	1.14 (1.05 - 1.25)	0.003	0.027
Alpha	14,439	829	1.21 (1.12 - 1.29)	<0.001	0.027
Delta	16,948	1,169	1.07 (1.01 - 1.14)	0.020	0.027
Omicron	929	101	0.94 (0.77 - 1.16)	0.578	0.027

Abbreviations: HR, Hazard Ratio; CI, Confidence Interval

[a] Based on a Cox Proportional Hazards model stratified by age and adjusted for confounders (sex, parental neurology, Charlson Comorbidity Index, employment status, income, and highest level of education)

Overview of ICD-8, ICD-10, and ATC codes

Supplementary Table 18: Neurological disorders categorized by ICD-8, ICD-10, and ATC codes

Neurological disorder	ICD-8	ICD-10	ATC
**Any neurological disorder**	All the below	All the below	All the below
**Parkinson's disease**	342	G20-G26	N04
**Neurodegenerative**	290	G30-G32	..
**Dementia**	290, 293.09, 293.19	F00-F03, G30.0-30.9, G31.9, G31.8	N06DX01, N06DA02, N06DA03, N06DA04
**Alzheimer's disease**	290.09, 290.10, 290.19	F00	..
**Vascular Dementia**[2]	293.09, 293.19	F01	..
**Immune-mediated**	340, 341, 733.09, 354.00 [a]	G35-G37, G70.0, G61.0 [a]	..
**Multiple Sclerosis (MS)**	340	G35	..
**Guillain Barré**	354.00 [a]	G61.0[a]	..
**Other immune-mediated**	341, 733.09	G36, G37, G70.0	..
**Epilepsy**	345 excluding 345.29	G40	N03
**Headache**	346, 791	G43, G44	N02C
**Narcolepsy**	347.00, 347.09	G47.4	..
**Nerve/nerve root and plexus disorders**	351-352, 355-358	G50-G59	..
**Polyneuropathy**	357-538, 354.00 [a], 734.90, 734.19, 391, 393, 712	G60-G64[a], M35.0, M32, M05-M06, M08.0	..
**Neuromuscular disease**	330	G70-G73	..
**Myopathy**	..	G72	..
**Other neuromuscular disease**	330	G70, G71, G73	..
**Cerebrovascular disease**	433-434	I60-69	..
**Central Nervous System (CNS) infections**[3]	013, 027.01, 036.09, 040-043, 045-046, 052.01, 053.02, 054.03, 055.01, 056.01, 062-065, 072.02, 075.01, 079.29,090.49, 094.9, 320, 322, 324, 392, 474	A066, A17, A321, A390, A521-A523, A80-A89, B003-B004, B010-B011, B020-B021, B050-B051, B060, B261-B262, B375, B451, B582, E236A, G00-G03, G060A-L, G061-G062, G079A-B, G079J-K	..
**Viral CNS infections**	040-043.99, 045-046, 052.01, 053.02, 054.03, 055.01, 056.01, 062-065, 072.02, 075.01, 079.29, 474	A80-A89, B003-B004, B010-B011, B020-B021, B050-B051, B060, B261-B262, G020	..
**Other neurological disorders**	321, 323, 331, 332, 333, 343, 344, 348, 349, 350, 353	G04, G05, G060M, G079C, G079F, G079G, G08, G09, G1, G8, G9	..

Table description: ICD-8 codes were used to exclude pre-existing neurological disorders and to adjust for parental neurological history. ICD-10 codes were used to exclude pre-existing neurological disorders, adjust for parental neurological history, and define outcome diagnoses. ATC codes were used in sensitivity analysis to identify individuals who had redeemed a prescription for neurological medication (see Supplementary material section on Sensitivity Analysis). Note, that the ICD-10 codes defined in the table extend beyond the G-chapter and include also other disorders treated in neurological inpatient and outpatient settings, including emergency room visits.

[a] The codes for Guillain-Barré (ICD-8: 354.00, ICD-10: G61.0) are included in both the immune-mediated disorders and the polyneuropathy disease category.

Supplementary Table 19: Charlson Comorbidity Index categorized by ICD-8 and ICD-10 codes

Disorder within CCI	ICD-8	ICD-10
**Myocardial infarction**	410	I21-I23
**Congestive heart failure**	42709, 42710, 42711, 42719, 42899, 78249	I50, I110, I130, I132
**Peripheral vascular disease**	440, 441, 442, 443, 444, 445	I70-I74, I77
**Cerebrovascular disease**	430, 440	I60-I69, G45-G46
**Dementia**	29009, 29020, 29309	F00-F03, F051, G30
**Chronic pulmonary disease**	430, 440, 515, 520	J40-J47, J60-J67, J684, J701, J703, J841, J920, J961, J982, J983
**Rheumatic disease (CTD)**	712, 716, 734, 446, 153.99	M05-M06, M09-M08, M30-M36, D86
**Peptic ulcer disease**	530.91, 530.98, 531, 535	K221, K25-K28
**Mild liver disease**	571, 573.01, 573.04	K700-K703, K709, K71, K73-K74, K760
**Diabetes without chronic complication (DIAB)**	571, 573.01, 573.04	E100-E101, E109-E111, E119
**Diabetes with chronic complication (DIAB_O)**	249.01-249.05, 249.08, 250.01-250.05, 250.08	E102-08, E112-E118
**Hemiplegia or paraplegia**	344	G81, G82
**Renal disease**	403, 404, 580, 581, 582, 583, 584, 590.09, 593.19, 753.1, 792	I12, I13, N00-N05, N11, N14, N17-N19, Q61
**Any malignancy, including lymphoma and leukaemia, except malignant neoplasm of skin**	204-207, 200-203, 275.59	C91-C95, C81-C85, C88, C90, C96
**Moderate or severe liver disease**	700.0, 700.2, 700.4, 700.6, 700.8, 573.00, 456.0	B150, B160, B162, B190, K704
**Metastatic solid tumour**	140, 195-199	C00-C80
**AIDS/HIV**	798.3	B21-B24

Abbreviations: CCI, Charlson Comorbidity Index

Table description: Note, some of the neurological diagnoses were part of Charlson Comorbidity Index (CCI), but since individuals with prior diagnoses of any neurological conditions were excluded from the study population, the neurological disorders within CCI were in practice not included in the model, and the CCI that was adjusted for throughout the paper consisted of non-neurological disorders only.

Supplementary Table 20: Definition of Exposures

	Definition	Comment
Confirmed SARS-CoV-2 infection (COVID-19-positive)	Positive SARS-CoV-2 polymerase chain reaction (PCR) by nasopharyngeal/tracheal test result	
COVID-19-negative	Negative SARS-CoV-2 polymerase chain reaction (PCR) by nasopharyngeal/tracheal test result	
SARS-CoV-2 reinfection/COVID-19 relapse	Positive SARS-CoV-2 PCR test after a minimum of 60 days since the last positive test.	
ICU admission	Admitted to ICU with procedure codes of either intensive care observation or intensive care treatment (NABE, NABB)	As defined in previous studies[78] and validated with a positive predictive value of 87.2%.[9] Note: Not conditioning on the duration of admission.
Inpatient at hospital	Admitted to the hospital as an inpatient (with a duration of at least 12 hours or ICU admission)	
Inpatient at a hospital with COVID-19	Admission as an inpatient in the hospital either a) with a COVID-19 diagnosis code (DB342, DB972A) or b) (-2, 14) days after a positive PCR test.	The individual was moved from reference to exposure group on the date where both criteria were met, i.e., inpatient, and positive COVID-19 test within time frame to avoid conditioning on future events.
ICU admission with COVID-19 infection	ICU admission either a) with a COVID-19 diagnosis code (DB342 or DB972A) or b) (-2, 14) days after a positive PCR test.	Same as above.

Abbreviations: ICU, Intensive Care Unit

Supplementary Table 21: Anti-infective agents categorized by ATC codes[10]

Type of anti-infective agent	ATC	Comment
**Antibacterial**		Including antiseptics
Anti-infectives	J01A-G, J01M, J01R, J01X	
Mycobacterium	J04A-B	Treatment of tuberculosis and lepra
Respiratory System	R02AA-AB	Antiseptics and antibiotics
**Antiviral**	J05A	Direct acting antivirals
**Antimycotic**	J02AA-AC, J02AX	

Table description: ATC codes used to identify individuals who have redeemed a prescription for any anti-infective agent.

Supplementary Table 22: Infection codes categorized by ICD-10 codes

Type of infection	ICD-10
**Any pulmonary infection (excl. COVID-19)**	J00-J06, J09-18, J20-22
Influenza	J09-11
Bacterial pneumonia	J12-18
Other pulmonary infection	J00-J06, J20-22

Table description: ICD-10 codes used to identify individuals hospitalised for any non-COVID-19 pulmonary infection.

Supplementary Table 23: Comorbidities related to neurological disorders and COVID-19 categorized by ICD-8 and ICD-10 codes

Type of Disease	ICD-8	ICD-10
**Heart disease**[4]	390-429	I00-I25, I27, I30-52
**Autoimmune disease**[5]		
**Pernicious anaemia**	281.0	D51.0
**Autoimmune haemolytic anaemia**	283.90–91	D59.1
**Idiopathic thrombocytopenic purpura**	446.49	D69.3
**Thyrotoxicosis**	242.00	E05.0
**Autoimmune thyroiditis**	245.03	E06.3
**Type 1 diabetes**	249	E10
**Primary adrenocortical insufficiency**	255.1	E27.1
**Multiple sclerosis**	340	G35
**Guillain-Barré syndrome**	354	G61.0
**Myasthenia gravis**	733.09	G70.0
**Iridocyclitis**	364	H20
**Crohn's disease**	563.01	K50
**Ulcerative colitis**	563.19	K51
**Autoimmune hepatitis**	571.93	K73
**Primary biliary cirrhosis**	571.90	K74.3
**Celiac disease**	269.00	K90.0
**Pemphigus**	694 (×694.05)	L10
**Pemphigoid**	694.05	L12
**Psoriasis vulgaris**	696.09–10, 696.19	L40 (×L40.4)
**Alopecia areata**	704.00	L63
**Vitiligo**	709.01	L80.9
**Seropositive rheumatoid arthritis**	712.19, 712.39, 712.59	M05–M06
**Juvenile arthritis**	712.09	M08
**Wegener's granulomatosis**	446.29	M31.3
**Polymyalgia rheumatica**	446.30–31, 446.39	M31.5–6, M35.3
**Systemic lupus erythematosus**	734.19	M32.1, M32.9
**Dermatopolymyositis**	716	M33
**Scleroderma**	734.0	M34
**Sjogren's syndrome**	734.90	M35.0
**Ankylosing spondylitis**	712.49	M45.9
**Trauma**[6]		
**Head trauma**	800, 801, 803, 850.99, 851-854	S02.0, S02.1, S02.7, S02.9, S06
**Fractures not involving the skull or spine**	807-809, 810-829	S12.8, S22.2-S22.5, S22.8, S22.9, S32.3-S32.5, S42.0-S42.7, S52, S62, S72, S82, S92, T10, T12

Table description: ICD-8 and ICD-10 codes were used in sensitivity analysis to identify individuals with any pre-existing comorbidity not included in Charlson Comorbidity Index (see Supplementary material section on Sensitivity Analysis).

Supplementary Methods

Statistical analysis:

We reported Hazard Ratios (HRs) including 95% CIs based on the Wald statistic. The Cox proportional hazard assumption was assessed with visual inspections of the Schoenfeld residuals. Statistical analyses were done in R, version 4.1.3 with the *survival* package, and the statistical significance was set to a two-sided P value < 0.05.

Analysis on: Time since positive SARS-CoV-2 test

Time since positive SARS-CoV-2 test was defined as a time-varying variable, where individuals changed exposure groups after a given amount of time. E.g., an individual followed for 5 months after a positive SARS-COV-2 test until censoring was in the <1 month group for the first month. After 1 month the individual changed to the 1-2 months group, where the individual was until 3 months after positive test. Then the individual changed to the 3-5 months group until the 5 months had passed, where the individual was censored.

Analysis on: Number of admissions (readmissions)

The number of admissions with COVID-19 was identified in a time-varying manner. All individuals were initially in the *No admissions with COVID-19* group. In case of a first admission with COVID-19 they changed to the *one admission with COVID-19* group until a potential re-admission with COVID-19, where they changed to the *two or more admissions with COVID-19* group. A third admission with COVID-19 did not lead to a change exposure groups.

Analysis on: Number of days in hospital (duration of admission)

The number of days in hospital during admission with COVID-19 was identified in a time-varying manner. As in the analysis on number of admissions, all individuals were initially in the *No admissions with COVID-19* group. In case of a first admission with COVID-19 they started in the *1-2 bed days* in hospital with COVID-19, and during admission they gradually changed groups. E.g., an individual in hospital for 5 days until censoring started in the *1-2 bed days* group and on the start of the third day, they changed to the *3-6 bed days* group until 5 days had passed, where they were censored.

Analysis on: Positive SARS-CoV-2 test compared with out-of-hospital infections:

Individuals with a prescription for any anti-infective agent between January 2019, and February 2020, year were excluded. Individuals with both a positive SARS-CoV-2 test and a prescription for any anti-infective agent were allocated to the SARS-CoV-2 positive exposure group.

Analysis on: Hospitalisation with COVID-19 compared with hospital-treated pulmonary infections:

In this analysis, individuals with any hospital-treated pulmonary infection between January 2010, and February 2020, were excluded. Individuals admitted with both COVID-19 and non-COVID-19 infections were allocated to the COVID-19 exposure group.

STROBE Statement

STROBE Checklist: Items that should be included in reports of cohort studies

	Item No	Recommendation	Page no.
**Title and abstract**	1	(*a*) Indicate the study's design with a commonly used term in the title or the abstract	1
		(*b*) Provide in the abstract an informative and balanced summary of what was done and what was found	2
**Introduction**			
Background/rationale	2	Explain the scientific background and rationale for the investigation being reported	3
Objectives	3	State specific objectives, including any prespecified hypotheses	4
**Methods**			
Study design	4	Present key elements of study design early in the paper	12
Setting	5	Describe the setting, locations, and relevant dates, including periods of recruitment, exposure, follow-up, and data collection	12-13
Participants	6	(*a*) Give the eligibility criteria, and the sources and methods of selection of participants. Describe methods of follow-up	12
		(*b*) For matched studies, give matching criteria and number of exposed and unexposed	NA
Variables	7	Clearly define all outcomes, exposures, predictors, potential confounders, and effect modifiers. Give diagnostic criteria, if applicable	12-15
Data sources/ measurement	8*	For each variable of interest, give sources of data and details of methods of assessment (measurement). Describe comparability of assessment methods if there is more than one group	12-15
Bias	9	Describe any efforts to address potential sources of bias	
Study size	10	Explain how the study size was arrived at	12
Quantitative variables	11	Explain how quantitative variables were handled in the analyses. If applicable, describe which groupings were chosen and why	14-15
Statistical methods	12	(*a*) Describe all statistical methods, including those used to control for confounding	13-15
		(*b*) Describe any methods used to examine subgroups and interactions	14-15
		(*c*) Explain how missing data were addressed	
		(*d*) If applicable, explain how loss to follow-up was addressed	NA
		(*e*) Describe any sensitivity analyses	15
**Results**			
Participants	13*	(a) Report numbers of individuals at each stage of study, e.g., numbers potentially eligible, examined for eligibility, confirmed eligible, included in the study, completing follow-up, and analysed	4
		(b) Give reasons for non-participation at each stage	NA
		(c) Consider use of a flow diagram	Figure 1
Descriptive data	14*	(a) Give characteristics of study participants (e.g., demographic, clinical, social) and information on exposures and potential confounders	Supplementary Table 1-2
		(b) Indicate number of participants with missing data for each variable of interest	Supplementary Table 1-2
		(c) Summarise follow-up time (e.g., average, and total amount)	Supplementary Table 1-2
Outcome data	15*	Report numbers of outcome events or summary measures over time	Supplementary Table 1-2

Main results	16	(*a*) Give unadjusted estimates and, if applicable, confounder-adjusted estimates and their precision (e.g., 95% confidence interval). Make clear which confounders were adjusted for and why they were included	Supplementary Sensitivity Analysis 1
		(*b*) Report category boundaries when continuous variables were categorized	X
		(*c*) If relevant, consider translating estimates of relative risk into absolute risk for a meaningful time period	NA
Other analyses	17	Report other analyses done, e.g., analyses of subgroups and interactions, and sensitivity analyses	8, Supplementary Sensitivity Analysis 1-6
**Discussion**			
Key results	18	Summarise key results with reference to study objectives	8
Limitations	19	Discuss limitations of the study, considering sources of potential bias or imprecision. Discuss both direction and magnitude of any potential bias	11
Interpretation	20	Give a cautious overall interpretation of results considering objectives, limitations, multiplicity of analyses, results from similar studies, and other relevant evidence	11
Generalisability	21	Discuss the generalisability (external validity) of the study results	8-11
**Other information**			
Funding	22	Give the source of funding and the role of the funders for the present study and, if applicable, for the original study on which the present article is based	Acknowledgements

*Give information separately for exposed and unexposed groups.

https://www.strobe-statement.org/checklists/

Supplementary References

1. Statens Serum Institut. Tidslinje for COVID-19. https://www.ssi.dk/-/media/arkiv/subsites/covid19/presse/tidslinje-over-covid-19/covid-19-tidslinje-for-2020-2022-lang-version---version-1---april-2022.pdf (2022).
2. Petersen, M. S., Lophaven, S. N., Weihe, P. & Lynge, E. High incidence of dementia in Faroese-born female residents in Denmark. *Alzheimer's & Dementia : Translational Research & Clinical Interventions* **6**, (2020).
3. Pedersen, E. M. J. *et al.* Infections of the central nervous system as a risk factor for mental disorders and cognitive impairment: A nationwide register-based study. *Brain, Behavior, and Immunity* **88**, 668–674 (2020).
4. Laursen, T. M., Munk-Olsen, T., Agerbo, E., Gasse, C. & Mortensen, P. B. Somatic Hospital Contacts, Invasive Cardiac Procedures, and Mortality From Heart Disease in Patients With Severe Mental Disorder. *Archives of General Psychiatry* **66**, 713–720 (2009).
5. Eaton, W. W., Pedersen, M. G., Nielsen, P. R. & Mortensen, P. B. Autoimmune diseases, bipolar disorder, and non-affective psychosis. *Bipolar disorders* **12**, 638–646 (2010).
6. Madsen, T. *et al.* Association Between Traumatic Brain Injury and Risk of Suicide. *JAMA* **320**, 580 (2018).
7. Reilev, M. *et al.* Characteristics and predictors of hospitalization and death in the first 11 122 cases with a positive RT-PCR test for SARS-CoV-2 in Denmark: a nationwide cohort. *International Journal of Epidemiology* **49**, 1468–1481 (2020).
8. Jacobsen, P. A. *et al.* Return to work after COVID-19 infection – A Danish nationwide registry study. *Public Health* **203**, 116–122 (2022).
9. Blichert-Hansen, L., Nielsson, M. S., Nielsen, R. B., Christiansen, C. F. & Nørgaard, M. Validity of the coding for intensive care admission, mechanical ventilation, and acute dialysis in the Danish National Patient Registry: a short report. *Clinical epidemiology* **5**, 9–12 (2013).
10. Köhler-Forsberg, O. *et al.* A Nationwide Study in Denmark of the Association between Treated Infections and the Subsequent Risk of Treated Mental Disorders in Children and Adolescents. *JAMA Psychiatry* **76**, 271–279 (2019).
