# Supplementary material for: Long-term neurological outcome after COVID-19 using all SARS-CoV-2 test results and hospitalisations in Denmark with 22-month follow-up
Source: Nat Commun. 2023 Jul 15;14:4235. doi: 10.1038/s41467-023-39973-6 (PMC10349860; doi:10.1038/s41467-023-39973-6)
Supplement: Supplementary file 3 — Reporting Summary [file 41467_2023_39973_MOESM3_ESM.pdf]

nature portfolio

Corresponding author(s): Michael E. Benros

Last updated by author(s): Jun 12, 2023

# Reporting Summary

Nature Portfolio wishes to improve the reproducibility of the work that we publish. This form provides structure for consistency and transparency in reporting. For further information on Nature Portfolio policies, see our Editorial Policies and the Editorial Policy Checklist.

## Statistics

For all statistical analyses, confirm that the following items are present in the figure legend, table legend, main text, or Methods section.

| n/a | Confirmed | |
|---|---|---|
| ☐ | ☒ | The exact sample size ($n$) for each experimental group/condition, given as a discrete number and unit of measurement |
| ☐ | ☒ | A statement on whether measurements were taken from distinct samples or whether the same sample was measured repeatedly |
| ☐ | ☒ | The statistical test(s) used AND whether they are one- or two-sided<br>*Only common tests should be described solely by name; describe more complex techniques in the Methods section.* |
| ☐ | ☒ | A description of all covariates tested |
| ☐ | ☒ | A description of any assumptions or corrections, such as tests of normality and adjustment for multiple comparisons |
| ☐ | ☒ | A full description of the statistical parameters including central tendency (e.g. means) or other basic estimates (e.g. regression coefficient) AND variation (e.g. standard deviation) or associated estimates of uncertainty (e.g. confidence intervals) |
| ☐ | ☒ | For null hypothesis testing, the test statistic (e.g. $F$, $t$, $r$) with confidence intervals, effect sizes, degrees of freedom and $P$ value noted<br>*Give P values as exact values whenever suitable.* |
| ☒ | ☐ | For Bayesian analysis, information on the choice of priors and Markov chain Monte Carlo settings |
| ☒ | ☐ | For hierarchical and complex designs, identification of the appropriate level for tests and full reporting of outcomes |
| ☐ | ☒ | Estimates of effect sizes (e.g. Cohen's $d$, Pearson's $r$), indicating how they were calculated |

*Our web collection on statistics for biologists contains articles on many of the points above.*

## Software and code

Policy information about availability of computer code

| | |
|---|---|
| Data collection | Not applicable |
| Data analysis | All analyses and illustrations were made using R version 4.1.3. The survival package version 3.2-13 was used for analyses. |

For manuscripts utilizing custom algorithms or software that are central to the research but not yet described in published literature, software must be made available to editors and reviewers. We strongly encourage code deposition in a community repository (e.g. GitHub). See the Nature Portfolio guidelines for submitting code & software for further information.

## Data

Policy information about availability of data

All manuscripts must include a data availability statement. This statement should provide the following information, where applicable:

- Accession codes, unique identifiers, or web links for publicly available datasets
- A description of any restrictions on data availability
- For clinical datasets or third party data, please ensure that the statement adheres to our policy

The following databases were used: The Microbiology Database, the Danish National Patient Register, the Danish Psychiatric Central Research Register, the Danish Education Register, the Danish Income Register, and the Danish National Prescription Register. The data used in this study are available to Danish researchers after approval of access from the data accessor authorities.

## Human research participants

Policy information about studies involving human research participants and Sex and Gender in Research.

| | |
|---|---|
| Reporting on sex and gender | Analyses were adjusted for sex as assigned at birth, which was the information available in the data. |
| Population characteristics | The study population consisted of 4,888,615 individuals of whom 49.2% were females, and the mean age at start was 39.9 (SD 23.3) years). |
| Recruitment | All individuals in the population of Denmark without pre-existing neurological disorders were included, therefore there was no bias in relation to selection of study participants. |
| Ethics oversight | This study was approved by the Danish Data Protection Agency and the Danish Health and Medicine Authority. According to Danish legislation, no further ethical approval or informed consent is required for register-based studies. |

Note that full information on the approval of the study protocol must also be provided in the manuscript.

# Field-specific reporting

Please select the one below that is the best fit for your research. If you are not sure, read the appropriate sections before making your selection.

☒ Life sciences ☐ Behavioural & social sciences ☐ Ecological, evolutionary & environmental sciences

For a reference copy of the document with all sections, see nature.com/documents/nr-reporting-summary-flat.pdf

# Life sciences study design

All studies must disclose on these points even when the disclosure is negative.

| | |
|---|---|
| Sample size | To attain the most precise estimates, the entire population of Denmark without pre-existing neurological disorders were included. The study population consisted of 4,888,615 individuals of whom 675,961 tested positive for SARS-CoV-2 during the study period. |
| Data exclusions | Individuals with pre-existing neurological disorders were excluded as we only considered new onset neurological disorders. |
| Replication | The findings were not replicated as no external dataset with appropriate information is avaible. |
| Randomization | Participants were not allocated into experimental groups. |
| Blinding | Due to the study being observational, blinding was not possible. |

# Reporting for specific materials, systems and methods

We require information from authors about some types of materials, experimental systems and methods used in many studies. Here, indicate whether each material, system or method listed is relevant to your study. If you are not sure if a list item applies to your research, read the appropriate section before selecting a response.

### Materials & experimental systems

| n/a | Involved in the study | |
|---|---|---|
| ☒ | ☐ | Antibodies |
| ☒ | ☐ | Eukaryotic cell lines |
| ☒ | ☐ | Palaeontology and archaeology |
| ☒ | ☐ | Animals and other organisms |
| ☒ | ☐ | Clinical data |
| ☒ | ☐ | Dual use research of concern |

### Methods

| n/a | Involved in the study | |
|---|---|---|
| ☒ | ☐ | ChIP-seq |
| ☒ | ☐ | Flow cytometry |
| ☒ | ☐ | MRI-based neuroimaging |
