# Supplementary material for: Long-term neurological outcome after COVID-19 using all SARS-CoV-2 test results and hospitalisations in Denmark with 22-month follow-up
Source: Nat Commun. 2023 Jul 15;14:4235. doi: 10.1038/s41467-023-39973-6 (PMC10349860; doi:10.1038/s41467-023-39973-6)
Supplement: Supplementary file 2 — Peer Review File [file 41467_2023_39973_MOESM2_ESM.pdf]

nature portfolio

Peer Review File

Long-term neurological outcome after COVID-19 using all SARS-CoV-2 test results and hospitalisations in Denmark

Open Access This file is licensed under a Creative Commons Attribution 4.0 International License, which permits use, sharing, adaptation, distribution and reproduction in any medium or format, as long as you give appropriate credit to the original author(s) and the source, provide a link to the Creative Commons license, and indicate if changes were made. In the cases where the authors are anonymous, such as is the case for the reports of anonymous peer reviewers, author attribution should be to 'Anonymous Referee' followed by a clear attribution to the source work. The images or other third party material in this file are included in the article's Creative Commons license, unless indicated otherwise in a credit line to the material. If material is not included in the article's Creative Commons license and your intended use is not permitted by statutory regulation or exceeds the permitted use, you will need to obtain permission directly from the copyright holder. To view a copy of this license, visit http://creativecommons.org/licenses/by/4.0/.

Editorial Note: This manuscript has been previously reviewed at another journal that is not operating a transparent peer review scheme. This document only contains reviewer comments and rebuttal letters for versions considered at Nature Communications.

REVIEWERS' COMMENTS

Reviewer #1 (Remarks to the Author):

This well conducted study provides important insights into the occurrence of neurological disorders following COVID-19.
Following my initial review, I am happy that the authors have addressed my comments.

Point by point response to the reviewers

Manuscript #: NCOMMS-23-14755-T

Title: Long-term neurological outcome after COVID-19 using all SARS-CoV-2 test results and hospitalisations in Denmark

---

REVIEWER REPORTS

Reviewer Comments:

Reviewer #1:
Remarks to the Author:
Comment 1:

This well conducted study provides important insights into the occurrence of neurological disorders following COVID-19.

Following my initial review, I am happy that the authors have addressed my comments.

Authors' reply: We thank the reviewer for the thorough assessment of our paper and recognition of the importance of this study.

Reviewer #2:
None

Reviewer #3:
None
